# Widespread Horizontal Gene Transfer from Circular Single-stranded DNA Viruses to Eukaryotic Genomes

**DOI:** 10.1186/1471-2148-11-276

**Published:** 2011-09-26

**Authors:** Huiquan Liu, Yanping Fu, Bo Li, Xiao Yu, Jiatao Xie, Jiasen Cheng, Said A Ghabrial, Guoqing Li, Xianhong Yi, Daohong Jiang

**Affiliations:** 1 State Key Laboratory of Agricultural Microbiology, Huazhong Agricultural University, Wuhan 430070, Hubei Province, P R China; 2 The Provincial Key Lab of Plant Pathology of Hubei Province, College of Plant Science and Technology, Huazhong Agricultural University, Wuhan, 430070, Hubei Province, P R China; 3 Current Address: Purdue-NWAFU Joint Research Center, Northwest A&F University, Yangling, 712100, Shaanxi Province, P R China; 4 Department of Plant Pathology, University of Kentucky, 201F Plant Science Building, 1405 Veterans Drive, University of Kentucky, Lexington, KY 40546-0312, USA

## Abstract

**Background:**

In addition to vertical transmission, organisms can also acquire genes from other distantly related species or from their extra-chromosomal elements (plasmids and viruses) via horizontal gene transfer (HGT). It has been suggested that phages represent substantial forces in prokaryotic evolution. In eukaryotes, retroviruses, which can integrate into host genome as an obligate step in their replication strategy, comprise approximately 8% of the human genome. Unlike retroviruses, few members of other virus families are known to transfer genes to host genomes.

**Results:**

Here we performed a systematic search for sequences related to circular single-stranded DNA (ssDNA) viruses in publicly available eukaryotic genome databases followed by comprehensive phylogenetic analysis. We conclude that the replication initiation protein (Rep)-related sequences of geminiviruses, nanoviruses and circoviruses have been frequently transferred to a broad range of eukaryotic species, including plants, fungi, animals and protists. Some of the transferred viral genes were conserved and expressed, suggesting that these genes have been coopted to assume cellular functions in the host genomes. We also identified geminivirus-like and parvovirus-like transposable elements in genomes of fungi and lower animals, respectively, and thereby provide direct evidence that eukaryotic transposons could derive from ssDNA viruses.

**Conclusions:**

Our discovery extends the host range of circular ssDNA viruses and sheds light on the origin and evolution of these viruses. It also suggests that ssDNA viruses act as an unforeseen source of genetic innovation in their hosts.

## Background

In addition to vertical transmission and gene acquisition from other distantly related species via horizontal gene transfer (HGT), organisms can also capture genetic material from extra-chromosomal elements (plasmids and viruses) during evolution. It is widely accepted that phages represent substantial forces in prokaryotic evolution, with the integrated phages (prophages) accounting for as much as 10-20% of some bacterial genomes [1,2]. In eukaryotes, animal retroviruses, which can integrate into host genome as an obligate step in their replication strategy, comprise approximately 8% of the human genome in the form of inherited endogenous retroviruses [3]. Moreover, the integrated retroviral genes have been demonstrated to play critical role in mammalian reproduction [4,5]. Recent data reveal that several non-retroviral viruses have also contributed to the genetic makeup of many eukaryotic organisms [6-15]. Especially, genes derived from ancestral nudiviruses have been co-opted to facilitate a parasitic lifestyle in parasitoid wasps [7]; and a gene derived from partitiviruses was exapted to regulate the activities of the phytohormone auxin, indole-3-acetic acid (IAA) in *Arabidopsis thaliana *[12]. Still, this type of transfer is thought to be rare in eukaryotes.

Viruses with circular single stranded DNA (ssDNA) genomes are the smallest viruses known to infect eukaryotes and are currently grouped into four families: *Anelloviridae, Circoviridae, Geminiviridae *and *Nanoviridae *(Virus Taxonomy: 2009, ICTV, http://www.ictvonline.org/virusTaxonomy.asp?version = 2009). The members of the first two families infect vertebrates and of the last two families infect plants. Recently a virus distantly related to circoviruses carrying a covalently closed circular, partially double-stranded ssDNA genome has been found to infect the marine diatom *Chaetoceros salsugineum* [16]. A similar virus was also discovered in *C. debilis *[17]. Moreover, recent viral metagenomic studies have shown that small circular ssDNA viruses are more prevalent and diverse in the environment than previously recognized [18-22].

Small circular ssDNA viruses commonly replicate their genomes in the nuclei of infected cells via a rolling circle replication (RCR) mechanism initiated by virus-encoded replication initiation protein (Rep), and there are clear similarities among the sequences of these proteins [23,24]. So far, no associated integrase activity has been identified for these viruses. However, Bejarano et al [25] reported multiple repeats of geminiviral Rep DNA that have been integrated into the nuclear genome of tobacco. In addition, Rep-like genes were also found in genomes of the parasitic protozoan *Entamoeba histolytica *and *Giardia intestinalis *[26]. These discoveries suggest that the small circular ssDNA viruses could also contribute to the genetic heritage of eukaryotic organisms. Considering that the circular ssDNA viruses are widespread in nature, the role played by these viruses in eukaryotic evolution needs to be evaluated.

Accordingly, we performed a systematic search for sequences related to known small circular ssDNA viruses in the publicly available eukaryotic genome databases. As our study was being prepared for publication, an independent group of investigators reported that sequences related to circoviruses were detected in the genomes of six vertebrate species [27]. Here we report our more comprehensive and convincing results based on sufficiently critical data analysis, bench research and phylogenetic analysis. Our studies have not only corroborated the integration of circovirus-related sequences in these six species, but they have also revealed that numerous sequences related to circoviruses, geminiviruses and nanoviruses have been integrated into the germlines of diverse eukaryotes including plants, fungi, animals and protists. Furthermore, we have demonstrated some of these integrated genes were conserved and expressed in eukaryotic organisms. In addition, we also identified geminivirus-like and parvovirus-like transposable elements in the genomes of fungi and lower animals, respectively. The origin and evolution of small circular ssDNA viruses were also discussed.

## Results and discussion

### Identification of circular ssDNA viral Rep-related proteins in eukaryotic systems

Rep proteins are commonly encoded by mobile elements (most phages and eukaryotic ssDNA viruses, some plasmids of Gram-positive bacteria, eukaryotic *helitron *transposons, etc.) but without cellular homologs and therefore have been recognized as virus/plasmid-specific proteins (hallmark proteins) [28,29]. The Rep proteins of eukaryotic ssDNA viruses contain RCR catalytic domain and a C-terminal NTPase/helicase domain [30,31]. With such structure, the sequence of the Rep protein of geminiviruses readily detected those of the geminivirus Rep catalytic domain (Gemini_AL1), central domain (Gemini_AL1_M) and the RNA helicase domain (RNA_helicase) by using the NCBI Conserved Domain Database searches (http://www.ncbi.nlm.nih.gov/Structure/cdd/wrpsb.cgi) (see Additional file 1: Figure S1). Likewise, the Rep of nanoviruses and circoviruses detected the putative viral replication protein domain (Viral_Rep) and the RNA helicase domain. On the other hand, the Rep of pLS1 family of prokaryotic plasmids [32] comprises only plasmid replication protein domain (Rep_2). Recently, the plasmid Rep containing an additional helicase domain has also been reported in a phytoplasma [33].

To investigate sequences closely related to Rep proteins of eukaryotic circular ssDNA viruses in other systems, we used the representative Rep proteins of geminiviruses and circoviruses to search against NCBI non-redundant (NR) protein database by PSI-BLAST [34]. After removing the known eukaryotic circular ssDNA viruses and subsequent reverse BLAST comparisons, we obtained a final dataset containing 113 Rep-like protein sequences from plasmids, other eukaryotic viruses and cellular genomes respectively (see Additional file 2: Tabular data S1). Most of these belonged to bacterial plasmids (typically phytoplasmal plasmids) and bacterial genomes. Conserved Domain searches showed that, apart from the known Rep_2 plus RNA_helicase domains, Rep proteins from bacterial plasmids had two other types of domain arrangements: Gemini_AL1 plus RNA_helicase and Viral_Rep plus RNA_helicase (see Additional file 1: Figure S1 and Additional file 2: Tabular data S1). The domain organization of Rep-like proteins from bacterial genomes showed high similarity to those of plasmids suggesting that these cellular homologs may originate from their plasmid counterparts.

**Figure 1:**
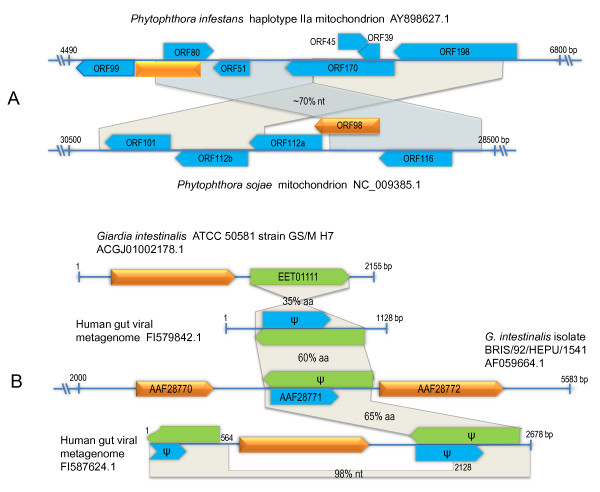
**Integrated plasmid or virus-like genes in *Phytophthora *sp**. (A) and *Giardia intestinalis *(B). Arrowhead boxes indicate ORFs (orange, Rep-like genes; other colors, unknown genes). Gray sectors connect corresponding homologous regions and the % nucleotide (nt) or amino acid (aa) identity are indicated. The annotated ORF names or accession numbers are indicated. ψ, interrupted ORF.

One apparently truncated Rep-like protein from the mitochondrion of oomycete *Phytophthora sojae *[35] contained Gemini_AL1 domain. Interestingly, we also found its coordinates in mitochondrion type II haplotypes of *P. infestans *[36] (Figure 1A), which has not been identified in previous reports. The mitochondrial regions containing the Rep-like sequences were absent in mitochondrion of *P. ramorum *and the type I haplotypes of *P. infestans *[36]. In addition, they are most closely related to the Reps of plasmids from the red algae *Porphyra tenera *[37]. These findings suggest that the Rep-like sequences are most likely derived from an integrated plasmid. Two circovirus Rep-related sequences have been found in the genome of canarypox virus [38] but not in other poxviruses, thus suggesting that they were acquired horizontally.

Our dataset also included the previously reported Rep-like genes in the genome of *G. intestinalis *isolate BRIS/92/HEPU/1541 and *E. histolytica *HM-1:IMSS [26]. These genes have been shown to be present in *G. intestinalis *ATCC 50581 strain GS/M H7 but not present in the *G. intestinalis *ATCC 50803 WB genome [39]. We found that two sequences of human gut viral metagenome from Genomic Survey Sequence (GSS) database share sequence similarities not only with the Rep-like genes of *G. intestinalis *but also with their neighboring genes (Figure 1B). This could provide evidence that these genes were originated from integrated proviruses. In addition to the known Rep-like genes in genomes of *E. histolytica *and *G. intestinalis*, we also identified 20 new Rep-like genes in genomes of other protozoan species as well as fungi, placozoans, and roundworms (see Additional file 2: Tabular data S1). Among these, the fungal Rep-like proteins contained the geminivirus-like domain, while the rest have domain similarities with Reps of nanoviruses or circoviruses. These findings suggest that the eukaryotic circular ssDNA viral genes may be of widespread occurrence in their host genomes but have yet to be discovered.

### Identification and validation of eukaryotic endogenous circular ssDNA virus-like sequences in germline genomes

To conduct this survey, we performed a comprehensive BLAST searches using as queries the viral Rep-like proteins in eukaryotic genomes and the protein sequences of representative eukaryotic circular ssDNA viruses against the genomic assemblies of 209 eukaryotes plus other uncompleted eukaryotic genomes in HTGS, WGS and GSS databases. This process identified 305 significant matches to Rep or CP proteins of geminiviruses, nanoviruses and circoviruses (Table 1 and Additional file 2: Tabular data S2). The most abundant of these virus-like sequences were related to the Rep genes, while only three sequences (one geminivirus-like, two circovirus-like) were related to CP genes, which were detected in tobacco and sloth genomes respectively. This finding is consistent with previous metagenomic research; possibly due to the fact that the Rep gene is more conserved during evolution. However, the possibility that possession of Rep sequences may offer some selective advantage to the host species cannot be ruled out either.

**Table 1:** Numbers of endogenous circular ssDNA virus-like sequences in eukaryotic genomes

Organism group	Organism	No. of virus-related genes	
		
		Rep	Capsid
Plants			
land plants	*Populus trichocarpa *(black cottonwood)	1	
	*Nicotiana tabacum *(common tobacco)		1
green algae	*Micromonas pusilla *(green algae) CCMP1545	1	
Fungi			
ascomycetes	*Aspergillus nidulans *FGSC A4	1	
	*Aspergillus fumigatus *A1163	1	
	*Aspergillus niger *CBS 513.88	1	
	*Trichoderma atroviride *IMI 206040	1	
	*Magnaporthe oryzae *70-15 (rice blast fungus)	1	
	*Nectria haematococca *mpVI 77-13-4	4	
	*Tuber melanosporum *Mel28 (Perigord truffle)	42	
basidiomycetes	*Laccaria bicolor *S238N-H82 (Bicoloured deceiver)	5	
Protists			
protozoans	*Entamoeba invadens *IP1	10	
	*Entamoeba terrapinae*	3	
	*Entamoeba histolytica *HM-1:IMSS	14	
	*Entamoeba dispar *SAW760	7	
	*Blastocystis hominis *Singapore isolate B (sub-type 7)	7	
	*Giardia intestinalis *ATCC 50581 strain GS/M H7	13	
	*Giardia intestinalis *isolate BRIS/92/HEPU/1541	2	
diatoms	*Phaeodactylum tricornutum *(diatom)	1	
Animals			
mammals	*Canis lupus familiaris *(dog) *	4	
	*Monodelphis domestica *(gray short-tailed opossum) *	1	
	*Felis catus*(domestic cat) *	6	
	*Ailuropoda melanoleuca *(giant panda) *	12	
	*Choloepus hoffmanni *(Hoffmann's two-fingered sloth) *		2†
gastropods	*Aplysia californica *(California sea hare)	1	
amphibians	*Xenopus (Silurana) tropicalis*(western clawed frog) *	2	
lancelets	*Branchiostoma floridae *(Florida lancelet) strain S238N-H82	7	
roundworms	*Brugia malayi *(agent of lymphatic filariasis)	1	
	*Loa loa *(African eyeworm)	10	
	*Wuchereria bancrofti *(agent of lymphatic filariasis)	3	
	*Onchocerca volvulus *(agent of onchocerciasis)	5	
crustaceans	*Lepeophtheirus salmonis *(salmon louse) strain Pacific	59	
mites & ticks	*Varroa destructor *(honeybee mite)strain Korean	56	
placozoans	*Trichoplax adhaerens *(placozoan) strain Grell-BS-1999	2	
hydrozoans	*Hydra magnipapillata *(hydrozoan) strain 105	18	

Total	35	302	3

* Some endogenous virus-like sequences from the indicated species have been reported by Belyi et al.[27]

† The two capsid-like sequences were incorrectly reported as Rep-like sequences by Belyi et al. [27]

**Figure 2:**
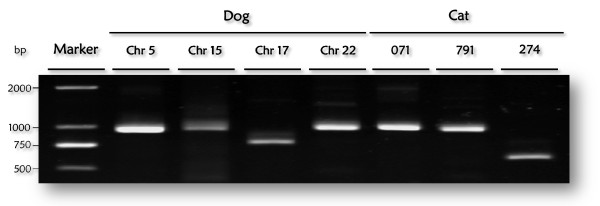
**Circovirus-like sequences in dog and cat genome were validated by PCR amplification and sequencing**. PCR products were fractionated by gel electrophoresis on 1% agarose gels and stained with ethidium bromide. Marker, DNA marker DL 2000. The sequences of bands of the expected sizes from lanes: Chr 5, Chr 22, 071, and 274 were deposited under Genbank accession numbers: JF414126-JF414131.

Because circular ssDNA viruses replicate their genomes in the nuclei of their host cells, sequence similarities between these viruses and host genomes could be attributed to trivial contamination of eukaryotic genomic DNA with viral sequences during cloning or sequence assembly. To rule out this possibility, we did a closer inspection of the raw sequence reads used for WGS assembly and the results indicated deep sequencing coverage across the junctions between the endogenous virus-like sequences and adjacent cellular sequences (see Additional file 2: Tabular data S2). These results suggest that the endogenous viral sequences were not artifacts of cloning or sequence assembly. For some eukaryotic genomes, the trace archives were not available, but their endogenous viral sequences underwent various degrees of degradation (see Additional file 2: Tabular data S2), suggesting that the viral sequences had invaded host genomes millions of years ago and therefore represent established germline infections. To validate these observations, we amplified and sequenced the endogenous circovirus-like sequences and their flanking host sequences from dog and cat tissues (Figure 2). The results revealed that the PCR products were of the expected sizes and the experimental sequences were identical to relevant regions of sequenced animal genomes.

Altogether, we discovered endogenous virus-like sequences in at least 35 species broadly distributed among nuclear genomes of plants, fungi, animals and protists. Remarkably, no anellovirus-like sequence was detected in any eukaryotic genome, although these viruses have been noted in various animal species [40].

### Characteristics and phylogenies of endogenous circular ssDNA virus-like sequences

**Figure 3:**
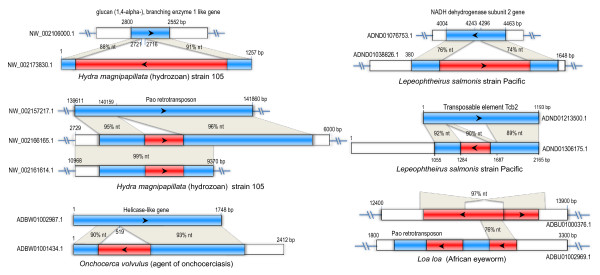
**Genomic comparisons showing the endogenous viral sequences inserted into coding regions of host genes**. Rectangular boxes with arrowheads indicate genes (Red, viral Rep-like genes; blue, host genes). Gray sectors connect corresponding homologous regions and the % nucleotide (nt) identity scores are indicated.

Compared to related exogenous viral genes, some endogenous virus-like sequences are full-length or near full-length genes while many others comprise only gene fragments. Despite pronounced sequence divergence, the conserved motifs of Rep protein can still be easily found in putative protein sequences of endogenous virus-like genes (see Additional file 1: Figure S2 and S3). The endogenous viral sequences are generally interspersed within non-coding regions of host genomes but several were found to be inserted into the coding regions of host genes or transposons (Figure 3). This finding suggests that these viral sequences have influenced host genome evolution through gene disruption.

**Figure 4:**
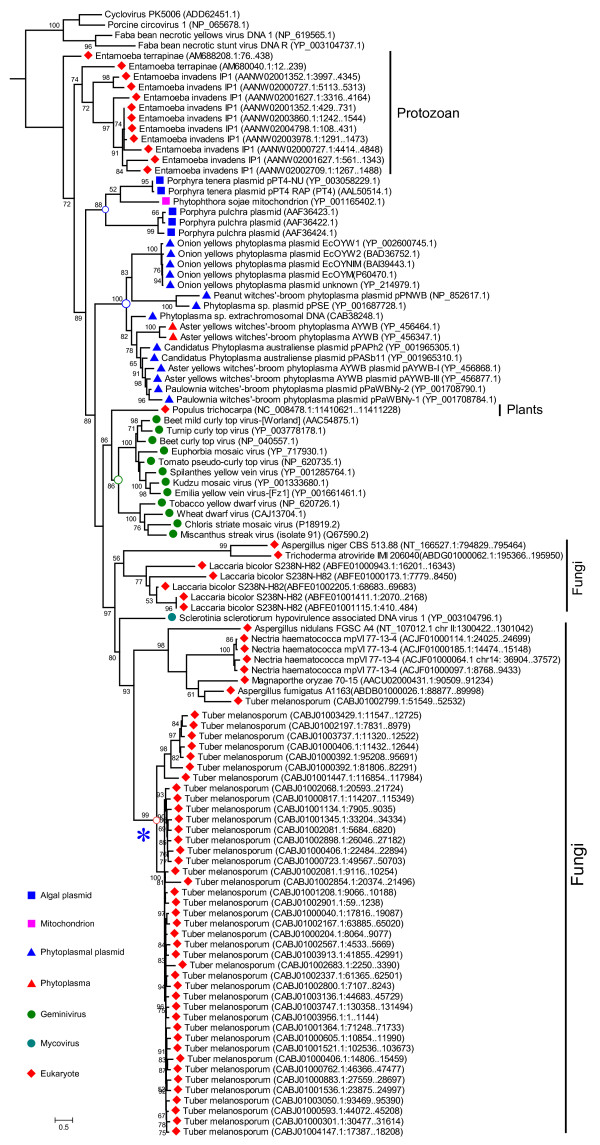
**Phylogeny of geminiviral Rep-like sequences from eukaryotes, known viruses, plasmids and phytoplasma**. The phylogenetic tree was built using PhyML-mixtures based on a multiple sequence alignment generated using COBALT with the Constraint E-value parameter setting to 0.1. This tree was rooted with circoviruses and nanoviruses. The topology of blue asterisk marked clade was evaluated independently. Only p-values of the approximate likelihood ratios (SH-test) > 0.5 (50%) are indicated. scale bars correspond to 0.5 amino acid substitutions per site. Sequence accession numbers are given for each sequence.

We next performed a comprehensive phylogenetic analysis to determine the relationship between the endogenous virus-like sequences and known circular ssDNA viruses as well as among endogenous sequences (Figure 4 and Additional file 1: Figure S4). As shown in Additional file 1: Figure S4, all the sequences formed three large clades: geminivirus-like, nanovirus-like and circovirus-like. In each clade, the endogenous virus-like sequences generally clustered distinctly with the known viruses but did not fall into the established viral families, suggesting that these virus-like sequences may have originated from previously undescribed circular ssDNA viral lineages. An exception was the only virus-like sequence detected in opossum (*Monodelphis domestica*) genome, which clustered within the *Circoviridae *clade and was most closely related to pig circoviruses.

Unlike Rep-like sequence in tobacco that were acquired more recently from members of *begomovirus *[41], one genera in the family *Geminiviridae*, the geminiviral Rep-like sequence in *Populus*, was located at the base of the *Geminiviridae *clade in the phylogenetic tree (Figure 4), suggesting that it was derived from integration of a *Geminiviridae *ancestor. Indeed, this sequence was degenerate, containing three inframe stop codons and one frameshift, an indication that it has been inserted a million years ago. Alternatively, it represents a distantly related geminiviral lineage infecting *Populus*.

All the virus-like sequences from fungi clustered together and were most closely related to the *Sclerotinia sclerotiorum hypovirulence associated DNA virus 1 *(SsHADV-1) (Figure 4), a mycovirus recently reported by us [42], suggesting that these endogenous viral sequences originated from SsHADV-1 like mycoviruses. Moreover, the SsHADV-1-like Reps were prevalent in viral metagenomes of different samples, including freshwater, human gut, rice paddy soil, marine environments and mosquito (see Additional file 1: Figure S5).

Our phylogenetic analysis also suggests that the circular ssDNA viruses were likely to co-evolve with their hosts over a long evolutionary time frame. For example, the virus-like sequences from lower eukaryotes (such as protozoans) were generally present at the base of each clade while those in relatively higher eukaryotes were more closely related to the known circoviruses, geminiviruses and nanoviruses that were infecting higher eukaryotes (see Additional file 1: Figure S4). There were, however, several exceptions, possibly due to horizontal viral transfers over short periods of time.

In most cases, the endogenous virus-like sequences from one species clustered together (such as those in salmon louse, honeybee mite, *Hydra *and roundworm species) (Additional file 1: Figure S4). Sequence comparison showed that, in each genome, some endogenous viral copies may have resulted from segmental duplication within host genomes after a single original integration, as similar levels of identity are observed between them as well as between their flanking genomic regions. While others may have been derived from multiple independent integration events, as no similarity was observed among their flanking genomic sequences (see Additional file 1: Figure S6).

Generally, the copy numbers of integrated viral sequences are less than 10 copies per species; whereas near sixty copies were identified in genomes of salmon louse (*Lepeophtheirus salmonis*) and honeybee mite (*Varroa destructor*) (see Additional file 1: Figure S4). Comparison of the viral copies and their adjacent host sequences in these two genomes showed that for most viral copies, no similarity was observed among their flanking genomic sequences, suggesting that most were derived from multiple invasions of same or very similar viruses. However, considering that the Rep protein of eukaryotic ssDNA viruses has DNA binding, endonuclease and NTPase activity required for viral DNA replication [30,31], the integrated genes encoding Rep-like proteins may catalyze their own single-strand excision and invasion, and therefore act as selfish genetic elements capable of parasite-like proliferation in the host genome. This scenario could be supported by the fact that the putative products of *Helitrons *[43], a eukaryotic rolling-circle transposon, shares motifs with the Reps of RCR plasmids and ssDNA viruses. Based on this fact, it has been suggested that ssDNA viruses might have evolved from RC transposons [43]. Our finding of endogenous viral Rep-like genes, however, favors the hypothesis that *Helitrons *may have arisen from ssDNA viruses which were integrated into the genome of an early eukaryotic ancestor [44].

### Identification of ssDNA virus-like transposable elements in eukaryotic genomes

**Figure 5:**
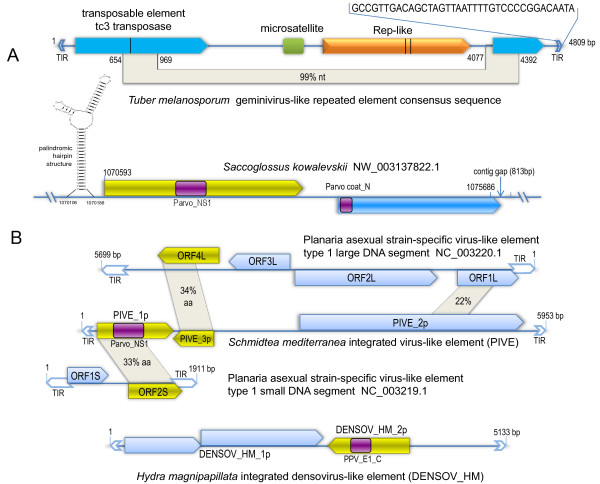
**Genomic organization of ssDNA virus-like transposons in fungi (A) and lower eukaryotes (B)**. (A) The genomic organization of geminivirus-like transposon in *Tuber melanosporum*. Arrowhead boxes indicate ORFs (orange, Rep-like gene; blue, transposase gene). The black vertical lines in the arrowhead boxes indicate stop codons. Green rectangular box indicates microsatellite sequence. The sequence of terminal inverted repeat (TIR) is shown at the top to the right. (B) The genomic organization and comparison of parvovirus-like transposon with related exogenous planaria virus. Yellow arrowhead boxes indicate Rep-like ORFs. Swallow tails indicate terminal inverted repeats (TIRs). The annotated ORF names are indicated. Purple rectangular boxes indicate protein domains and the domain family names are shown: Parvo_NS1, Parvovirus non-structural protein NS1 (pfam01057); Parvo_coat_N, Parvovirus coat protein VP1 (pfam08398); PPV_E1_C, Papillomavirus helicase (pfam00519). Gray sectors connect corresponding homologous regions and the % nucleotide (nt) or amino acid (aa) identity are indicated. The Planaria asexual strain-specific virus-like element has not been found to integrate in the host genome.

There are 42 geminivirus-like Rep genes or remnants interspersed in the genome of Perigord black truffle (*Tuber melanosporum*), an ectomycorrhizal fungus. All but one are most closely related to each other and formed a distinct clade (Figure 4). They share high (> 95%) nucleotide sequence identities with each other and thus allow us to reconstruct a consensus sequence. The reconstructed copy contains one interrupted Rep-like open reading frame (ORF), two transposase ORFs (one is interrupted and the other is truncated), and one microsatellite sequence (Figure 5A). It also contains 37-bp terminal inverted repeats (TIRs) but no obvious target site duplications (TSDs). It is most likely that this copy represents a novel transposon related to geminivirus identified in a eukaryotic genome. The genetic distances among these transposable repeats are very short suggesting that the transposons have undergone recent large-scale amplification in the host genome.

In addition to the geminivirus-like transposon, we have also identified a parvovirus (linear ssDNA)-like repetitive element in the acorn worm (*Saccoglossus kowalevskii*) genome (see Additional file 2: Tabular data S3). Like parvoviruses, this repetitive element contains two large ORFs: one putative ORF encodes a protein containing parvovirus non-structural protein NS1 domain (Parvo_NS, pfam01057) and the other putative ORF encodes a protein containing parvovirus coat protein VP1 domain (Parvo_coat_N, pfam08398) (Figure 5B). It also possesses a palindromic hairpin structure at its 5' terminal sequence, which is commonly found in parvoviruses. There are over 50 copies of this repeat interspersed in the genome. Some of these contained degraded ORFs; and some contained only a single ORF or a fragment. Furthermore, we also identified numerous parvovirus non-structural protein-like sequences in genomes of the hydrozoan *Hydra magnipapillata *and the planarian *Schmidtea mediterranea *(Figure 5B). We noted that these have been annotated as integrated virus-like element: DENSOV_HM and PIVE in Repbase Update, respectively [Jurka J, Repbase Reports 8(3), 182-182 (2008), http://www.girinst.org/2008/vol8/issue3/DENSOV_HM.html; Rebrikov DV et al. Repbase Reports 8(2), 166-166 (2008), http://www.girinst.org/2008/vol8/issue2/PIVE.html].

Phylogenetic analysis revealed that PIVE is more closely related to planarian (*Girardia tigrina*) virus, Planaria asexual strain-specific virus-like element (PEVE) (see Additional file 1: Figure S7), which has not yet been found to integrate in host genome [45]. The DENSOV_HM was located at the base of the papillomavirus clade and did not cluster within family *Papillomaviridae*. Furthermore, their genome structure is different. Therefore it may have originated from integration of the virus in new family infecting hydrozoan. The parvovirus-like sequences from acorn worm grouped together with PIVE_1p and PEVE small segment and these further clustered with parvoviruses. Considering that the genome structure of acorn worm repeated element is also similar to parvoviruses, it is most likely that it derived from parvovirus lineage infecting acorn worm distantly related to known parvoviruses.

Consequently, these findings provide direct evidence that eukaryotic transposons could originated from ssDNA viruses.

### Preservation and expression of endogenous viral genes in host genomes

**Figure 6:**
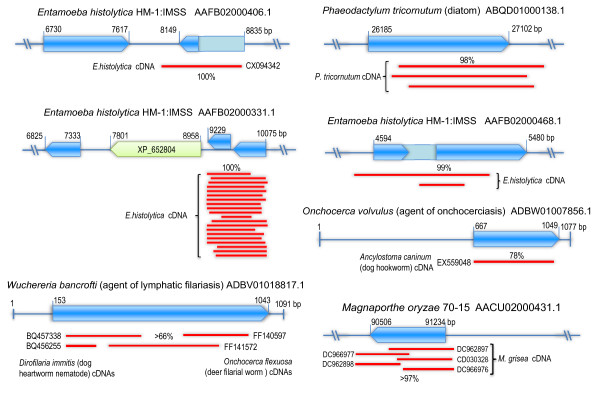
**Organization and transcription maps of endogenous viral Rep-like genes**. Blue arrowhead boxes indicate Rep-like ORFs. Similar regions of expressed sequences are identified and the % nt identity with endogenous viral sequences are indicated. Note that the actual endogenous viral sequence extended beyond the ORF in two contigs (AAFB02000406.1 and AAFB02000468.1) of *Entamoeba histolytica*.

Examination of the potential coding capacity of endogenous viral sequences indicates that most were truncated and degraded, containing numerous premature stop codons, frameshift mutations, and insertions or deletions (see Additional file 2: Tabular data S2), suggesting that these are unlikely to have functions. Some endogenous viral sequences however, were found to encode uninterrupted ORFs. The conservation of relatively long ORFs suggests that they may have evolved under functional constraints since integration. While intact ORFs could also reflect recent insertion rather than functional maintenance in a long-standing history within the host genome. Analysis of transcription products can provide stronger evidence of functional maintenance. Accordingly, we used putative amino acid sequences of endogenous viral sequences to search NCBI Expressed Sequence Tags (ESTs) database for the corresponding mRNAs with the tBLASTn program. Through subsequent sequence comparisons, it was clear that some endogenous viral sequences are expressed as mRNAs in host genomes because they share high sequence identity with most of their ESTs and adjacent host sequences over full-length sequences (Figure 6 and Additional file 2: Tabular data S4). Interestingly, ESTs related to endogenous viral sequences of the rice blast fungus *M. oryzae *and two roundworm species were found in related species whose genome sequences are not currently available, suggesting that there were similarly expressed insertions in these species. In fact, we have also amplified these virus-like sequences from different strains of the rice blast fungus and detected their expression (data not shown).

The fact that endogenous viral sequences are conserved and expressed in host organisms suggests that these viral genes have been coopted to assume cellular functions in eukaryotic genomes. It should be noted that expression of mRNA from endogenous viral sequences was also detected in the parasitic protozoan *E. histolytica*, although their long ORFs were defective (Figure 6). Perhaps selection to maintain these viral sequences has recently been lost.

We also detected endogenous viral sequence-related ESTs in some plants and animal species (see Additional file 2: Tabular data S5). Because the genome sequences of relevant host species are not available or available but not well matched with their ESTs, it remains to be established whether they represent authentic expressed endogenous viral sequences.

### The host range of circular ssDNA viruses

Circoviruses are previously known to infect only birds and pigs [46]. These viruses have been detected in dragonflies, fish and human most recently [47-49]. Geminiviruses and nanoviruses only have been found to infect higher plants [23,50]. Recent metagenomic studies uncovered that these circular ssDNA viruses were commonly found in various environmental samples, but it is difficult to provide information on the host range and ecology for these viruses.

The endogenous circovirus-like sequences in honeybee mite were most closely related to cycloviruses (Additional file 1: Figure S4), members of a recently proposed genus in the family *Circoviridae*, which were commonly found in faecal samples of human and chimpanzee by viral metagenomics [20]. In addition, the endogenous virus-like sequences from some species of various organisms (such as amphibians, algae, diatom, gastropod, etc.) were clustered with viral metagenomic sequences or circovirus-like genomes identified from environmental samples [18]. These findings suggest that these various species are the definitive hosts of relevant environmental viruses.

**Figure 7:**
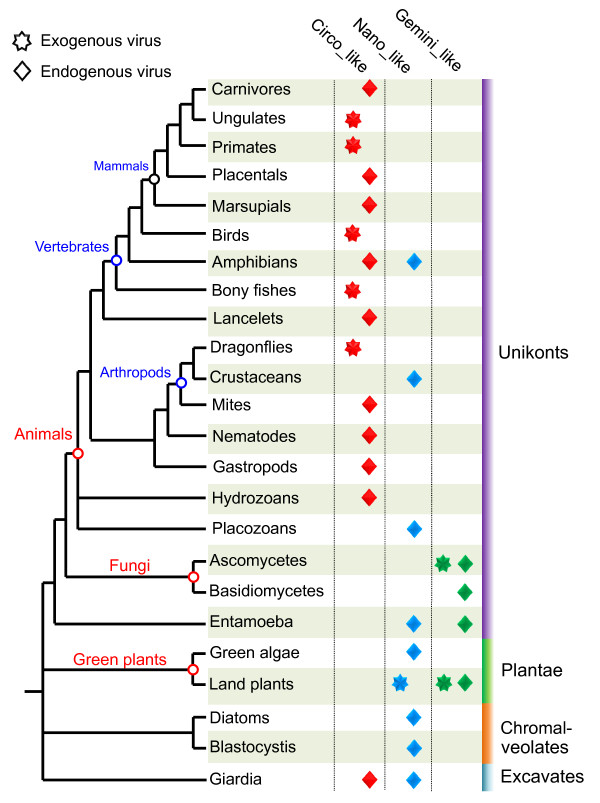
**A tree of eukaryotes showing the known distribution of endogenous viral-like sequences and exogenous circo-, nano- and geminivirus like viruses**. This tree was drawn base on The Tree of Life Web Project (http://tolweb.org/).

Altogether, we discovered endogenous ssDNA virus-like sequences in host species broadly distributed in four of the five supergroups of eukaryotes [51], namely Unikonta (including animals, fungi and *Entamoeba*), Plantae (including land plants and green algae), Chromalveolata (including diatoms and *Blastocystis*) and Excavata (including *Giardia*) (Figure 7). The endogenous viral sequences identified here as molecular fossil records of past viral invasions provide unambiguous definitive hosts for these viruses and extend the host range of circular ssDNA viruses.

Interestingly, although the endogenous circovirus/nanovirus-like sequences occurred widely in the genomes of eukaryotic species, ranging from unicellular organisms to mammals, we did not detect any of these sequences in plant, bird and pig genomes sequenced to-date. In contrast, the nematodes (roundworms) were not known to be infected by ssDNA virus, but endogenous circovirus-like sequences occurred in some nematode species. In addition, geminivirus-like sequences were found in some fungal genomes. So far, however no genetically related exogenous counterparts were found in these fungi, even though some of these, such as the rice blast fungus *Magnaporthe oryzae*, were widely studied. Likewise, *Populus *is not known to be infected by geminiviruses but harbored one endogenous geminiviral sequence. These observations suggest that some of the endogenous viral sequences could provide immune protection in the host similar to the endogenous retroviral capsid proteins in mice and sheep, which offer protection against exogenous retroviral infections [52,53].

### The origin and evolution of circoviruses and nanoviruses

Based on the different phylogenies between the N-terminal and the C-terminal regions of circovirus Rep, Gibbs and Weiller [54] suggested that circovirus Rep proteins may have evolved by a recombination event between the Rep protein of nanoviruses and an RNA binding protein encoded by picorna-like viruses after the nanoviruses switched hosts to infect a vertebrate. However, it seems unlikely that the virus recombination event took place in a vertebrate considering the fact that endogenous circovirus-like sequences were widely found in nonvertebrate species. To examine more thoroughly the origin and evolution of circoviruses and nanoviruses, we selected representative Rep-like proteins from viruses, plasmids and bacterial genomes and used sufficient samples to construct phylogenetic trees. In consideration of a possible recombination event, we aligned and performed phylogenetic analysis corresponding to full-length Rep genes, the N-terminal and C-terminal regions respectively. As shown in Additional file 2: Figure S8, circovirus-like sequences and viral Rep-like sequences from bacterial plasmid and bacterial genomes clustered together in all trees. However, while nanovirus-like sequences clustered with circovirus-like sequences in the N-terminal tree they were grouped with geminivirus-like sequences in the C-terminal tree. In the full-length Rep tree, nanovirus-like sequences were placed between the geminivirus-like and circovirus-like sequences, possibly due to the compromise of different phylogenetic signals from the two parts of nanovirus-like Reps. Therefore, if a recombination event had occurred, it is likely to have taken place in the nanovirus-like Reps rather than in the circovirus-like Reps.

It has been proposed that eukaryotic ssDNA viruses may have evolved from prokaryotic plasmids or phages [31]. In our phylogenetic trees, the virus-like sequences from bacterial plasmid and bacterial genomes were generally located at the base of circovirus-like sequences, suggesting that circoviruses might have originated from relevant bacterial plasmids. Considering that the nanovirus-like sequences clustered with circovirus-like sequences in the N-terminal tree, it is most likely that the nanoviruses shared the most recent common ancestor with circovirus-like viruses and subsequently the C-terminal sequences of ancestor nanoviral Reps may have recombined with those of geminivirus-like viruses or plasmids. But the possibility that nanovirus-like Reps were the result of convergent evolution cannot be ruled out. The Canarypox virus and the ancestor of picorna-like viruses may have captured the helicase domain sequences from circovirus-like viruses by recombination.

### The origin and evolution of geminiviruses

Based on the observations that geminiviruses occupied a common ecological niche with phytoplasmas and their Reps shared a most recent common ancestor with phytoplasmal plasmids in phylogenetic analysis, Krupovic et al [55] proposed that the geminiviruses may have originated from phytoplasmal plasmid followed by acquisition of the capsid gene from an ssRNA plant virus. However, in view of the recent reports on the geminivirus-like mycovirus and numerous related sequences in fungal genomes, the evolutionary relationships among these geminivirus-like elements need to be revaluated. To address this question, we constructed phylogenetic trees for the representative Rep-like proteins from plants, fungi, phytoplasma and algae using the full-length Rep genes, the N-terminal RCR catalytic domain and C-terminal helicase domain sequences respectively (Additional file 2: Figure S9). In all trees, the plant geminiviral Reps clustered together with fungal Reps, suggesting that they shared a more recent common ancestor with those from fungi rather than from phytoplasmal plasmids. Furthermore, although the Rep protein of SsHADV-1 is related to geminiviruses, the genome organization of SsHADV-1 and particle morphology is distinct from those of geminiviruses [42]. Although the capsid protein of SsHADV-1 lacks sequence similarity with those of any geminiviruses, its most similar sequences are commonly found in environmental samples. In addition, sequences related to SsHADV-1 were widely found in fungal genomes and diverse metagenomic samples. These results suggest that SsHADV-1 and related viruses from fungi and environment may have evolved independently rather than being descendent from geminiviruses or vice versa. Therefore, it is possible that the ancestor of geminiviruses and related fungal viruses may have occurred prior to the separation of plants and fungi, and subsequently perhaps they had a unique path to evolution in their hosts.

## Conclusions

Our study provided comprehensive and convincing evidence that the genes of small circular ssDNA viruses have been transferred into a broad range of eukaryotic genomes, and some of the transferred genes were also conserved and functional in host genomes. This discovery extends the host range of circular ssDNA viruses and offers insight into the origin and evolution of relevant viruses. Furthermore, our finding also revealed that the capture and functional assimilation of exogenous viral genes may represent an important force in the evolution of eukaryotes.

## Methods

### Genome screening

In order to screen for the circular ssDNA virus-related sequences in eukaryotic genomes, we performed tBLASTn searches against different NCBI sequence databases (http://blast.ncbi.nlm.nih.gov/Blast.cgi) using as queries the representative peptide sequences derived from viruses in families *Anelloviridae, Circoviridae, Geminiviridae *and *Nanoviridae*. NCBI databases used for sequence searches included nr (all GenBank + RefSeq Nucleotides + EMBL + DDBJ + PDB sequences + HTGS phase 3 but excluding HTGS phase 0,1,2, EST, GSS, STS, PAT, WGS), refseq_genomic (genomic entries from NCBI's Reference Sequence project), NCBI Genomes/chromosome (a database with complete genomes and chromosomes from the NCBI Reference Sequence project), wgs (a database for whole genome shotgun sequence entries), gss (Genome Survey Sequence, includes single-pass genomic data, exon-trapped sequences, and Alu PCR sequences), htgs (unfinished High Throughput Genomic Sequences: phases 0, 1 and 2), and the eukaryotes genomic BLAST database. All non-redundant matches from these searches with E-values ≤1e-5 were extracted along with 1 kb of flanking regions, and then were used to screen the non-redundant (NR) protein database using BLASTx. All genomic sequences from host genomes that unambiguously matched viral proteins were considered as candidate endogenous viral sequences. These candidate endogenous viral sequences were used to research the databases for other homologous sequences that would have been missed during initial searches using the known extant viruses. All database searches were performed online and were completed in June 2010.

### Examining possible chimeras or errors in assembling of endogenous viral sequences

To rule out the possibility that these endogenous viral sequences were chimeric clones or misassembled from contaminated sequences of exogenous incidental viral sequences, we searched against archival data of the eukaryotic genome sequencing using their endogenous viral sequences and flanking cellular sequences as megaBLAST queries on the NCBI Trace Archive (http://www.ncbi.nlm.nih.gov/blast/mmtrace.shtml) with the cut-off value: > 95% nt identity, respectively; and carefully examined the junctions between endogenous viral sequences and cellular sequences. The statistics of junction coverages that show the number of trace records containing the junctions between endogenous viral sequences and cellular sequences are listed in Additional file 2: Tabular data S2.

### Sequence comparison and phylogenetic analysis

The putative peptides of endogenous viral sequences were obtained according to BLASTx hits and manual editing. The in-frame stop codons were indicated as X. Multiple alignments of protein sequences were constructed either using MCOFFEE (when the number of sequences < 50) [56] or using COBALT [57] (http://www.ncbi.nlm.nih.gov/tools/cobalt/cobalt.cgi?link_loc=BlastHomeAd) and manually edited. To give the best alignment, the alignment parameter Constraint E-value and Word Size were adjusted for different datasets when using COBALT. Although many of the endogenous viral sequences are of different lengths in alignments, it is now well known that sequences of very different lengths can be accurately placed on phylogenies [58]. Hence, all the putative peptides of endogenous viral sequences were used for the phylogenetic analysis with proteins of representative exogenous viruses to determine the full-scale evolutionary relationships among them. Maximum likelihood (ML) phylogenies were estimated using amino acid sequence alignments with PhyML-mixtures [59,60], assuming the EX2 mixture model [60] and SPR tree topologies search strategy [61]. Gaps in alignment are systematically treated as unknown characters. The reliability of internal branches was evaluated based on approximate likelihood ratio test (aLRT) statistics [62].

### Detection of expression of endogenous viral sequences from host genomes

To investigate whether endogenous viral sequences could be expressed in host genomes, we first, used the endogenous viral sequences to screen the NCBI EST database using the method described in Genome screening. Subsequently, we used the identified virus-related ESTs to compare with host genomes and virus genomes by megaBLAST to determine whether they were expressed sequences from host genomes or the result of laboratory contamination.

### PCR amplification and DNA sequencing

Genomic DNA samples of dog (*Canis lupus familiaris*) and cat (*Felis catus*) were obtained from Zyagen Laboratories (USA). To PCR amplify the candidate DNA fragments from these DNA samples, primers pairs were designed based on the virus-like sequences and their flanking cellular sequences, see Additional file 3: Table S1 for the primers pairs used. PCR products were fractionated by gel electrophoresis on 1% agarose gels and stained with ethidium bromide. DNA was sequenced by Sanger methods at the Beijing Genomics Institute (BGI). New sequences generated in this study were deposited in the GenBank under accession numbers: JF414126-JF414131.

## List of abbreviations

aLRT: approximate likelihood ratio test; BLAST: Basic Local Alignment Search Tool; ESTs: Expressed Sequence Tags; CP: capsid protein; GSS: Genomic Survey Sequence; HTGS: High Throughput Genomic Sequence; HGT: horizontal gene transfer; ICTV: International Committee on Taxonomy of Viruses; NCBI: National Center for Biotechnology Information; ORF: open reading frame; PCR: Polymerase chain reaction; RCR: rolling circle replication; ssDNA: single-stranded DNA; SPR: subtree prune and regraft; TIRs: terminal inverted repeats; TSDs: target site duplications; Rep: replication initiator protein; TEs: transposable elements; WGS: Whole Genome Shotgun.

## Authors' contributions

HL, YF and DJ conceived and designed the study; HL performed the computational analyses and lab experiments; HL, YF, DJ, BL, XY, SAG, GL, JX, JC and XY analyzed data; and HL, YF, DJ and SAG wrote the paper. All authors read and approved the final manuscript.

## Supplementary Material

Additional file 1 **supplementary figures**. This file includes 9 supplementary figures. Figure S1 illustrates the domain organization of different Rep-like proteins. Figure S2 and S3 show multiple alignments of circovirus, nanovirus or geminivirus Rep-like sequences, respectively. Figure S4 shows the phylogeny of viral Rep-like sequences from eukaryotes, known viruses and viral metagenomes. Figure S5 shows the phylogeny of geminiviral Rep-like sequences in viral metagenomes. Figure S6 shows the alignment of viral insertion loci in a genome. Figure S7 shows the phylogeny of parvovirus-like transposons. Figure S8 and S9 show the phylogenies of full-length, N-terminal and C-terminal regions of circoviral or geminiviral Rep-like proteins, respectively. Click here for file

Additional file 2 **supplementary Tabular data**. This file contains 5 supplementary Tabular data. Tabular data S1 shows the characterization of eukaryotic circular ssDNA viral Rep-like proteins in other systems. Tabular data S1 gives results of all detected endogenous circular ssDNA virus-like sequences in this study. Tabular data S3 shows the endogenous parvovirus-like sequences in acorn worm genome. Tabular data S4 summarizes the expressed endogenous viral sequences. Tabular data S5 lists the circular ssDNA virus-related ESTs which have not been determined if they were expressed endogenous viral sequences. Click here for file

Additional file 3 **supplementary table S1**. This file lists primers used for PCR of endogenous virus-like regions of dog and cat genomes. Click here for file
