# Supplementary material for: Widespread Horizontal Gene Transfer from Circular Single-stranded DNA Viruses to Eukaryotic Genomes
Source: BMC Evol Biol. 2011 Sep 26;11:276. doi: 10.1186/1471-2148-11-276 (PMC3198968; doi:10.1186/1471-2148-11-276)
Supplement: Additional file 1 — supplementary figures. This file includes 9 supplementary figures. Figure S1 illustrates the domain organization of different Rep-like proteins. Figure S2 and S3 show multiple alignments of circovirus, nanovirus or geminivirus Rep-like sequences, respectively. Figure S4 shows the phylogeny of viral Rep-like sequences from eukaryotes, known viruses and viral metagenomes. Figure S5 shows the phylogeny of geminiviral Rep-like sequences in viral metagenomes. Figure S6 shows the alignment of viral insertion loci in a genome. Figure S7 shows the phylogeny of parvovirus-like transposons. Figure S8 and S9 show the phylogenies of full-length, N-terminal and C-terminal regions of circoviral or geminiviral Rep-like proteins, respectively. [file 1471-2148-11-276-S1.PDF]

Additional File 1

Supplementary figure legends:

Figure S1 Domain organization of Rep-like proteins from circular ssDNA viruses, plasmids and bacterial genomes.

The base map of domains was drawn using NCBI Conserved Domains searches (http://www.ncbi.nlm.nih.gov/Structure/cdd/wrpsb.cgi). Rep_2, Plasmid replication protein (pfam01719), Viral_Rep, Putative viral replication protein (pfam02407); Gemini_AL1, Geminivirus Rep catalytic domain (pfam00799); Gemini_AL1_M, Geminivirus rep protein central domain (pfam08283); RNA_helicase, RNA helicase (pfam00910). pLS1, Plasmid pLS1 (NP_040421); TYLCSV, *Tomato yellow leaf curl Sardinia virus* (CAA43466); FBNYV, *Faba bean necrotic yellows virus* (O39828); PCV2, *Porcine circovirus type 2* (AAQ94098); Bifidobacterium p4M, *Bifidobacterium pseudocatenulatum* VMKB4M plasmid p4M (NP_613078); Phytoplasma pOYM, Onion yellows phytoplasma plasmid pOYW (BAB39758); Phytoplasma pPASb11, *Candidatus Phytoplasma australiense* plasmid pPASb11 (YP_001965310); Algal plasmid, *Porphyra pulchra* plasmid (AAF36423); Clostridium saccharolyticum, *Clostridium saccharolyticum* WM1 (YP_003824178); Corynebacterium pseudogenitalium, *Corynebacterium pseudogenitalium* ATCC 33035 (ZP_03920710).

Figure S2 Multiple alignment showing the sequence length and conserved motifs of circovirus/nanovirus Rep-like sequences.

The alignment was obtained using the COBALT program with default parameters (http://www.ncbi.nlm.nih.gov/tools/cobalt/cobalt.cgi?link_loc=BlastHomeAd). Gray shaded numbers indicate present day circoviruses, nanoviruses and satellites. The names of representative ssDNA viruses and satellites as well as the selected endogenous circovirus/nanpvirus Rep-like sequences that correspond to the numbers are indicated at the bottom of the alignment. (A) The outline of alignment. Blocks in the protein alignment represent aligned aa residues and lines represent gaps or missing data. (B) Alignment details. The in-frame stop codons were indicated as X. The default color scheme for ClustalW alignment in the Jalview program was used. The regions of Rep conserved motifs are indicated.

Figure S3 Multiple alignment showing the sequence length and conserved motifs of geminivirus Rep-like sequences.

The alignment was obtained using the MCOFFEE program (http://tcoffee.vital-it.ch/cgi-bin/Tcoffee/tcoffee_cgi/index.cgi?stage1=1&daction=MCOFFEE::Advanced). Gray shaded numbers indicate present day mycovirus and geminiviruses. The names corresponding to the numbers are indicated at bottom of the alignment. (A) An outline of the alignment. Blocks in the protein alignment represent aligned aa residues and lines represent gaps or missing data. (B) Alignment details. The in-frame stop codons are indicated as X. The default color scheme for ClustalW alignment in the Jalview program was used. The regions of Rep conserved motifs are indicated.

Figure S4 Phylogeny of viral Rep-like sequences from eukaryotes, known viruses and viral metagenomes. The phylogenetic tree was built using PhyML-mixtures based on a multiple sequence alignment generated using COBALT with the Word Size parameter setting to 3. The base tree was drawn using Interactive Tree Of Life Version 1.9 (http://itol.embl.de/#). The geminivirus-like group was chosen as outgroup for presentation purposes. The p-values of approximate likelihood ratios (SH-test) plotted as circle marks on the branches (only p-values >0.5 are indicated) and circle size is proportional to the p-values. Branch colors correspond to taxonomic groups and the main taxonomic groups are indicated. Scale bars correspond to 0.1 amino acid substitutions per site. Sequence accession numbers are given for each sequence.

Figure S5 Phylogeny of geminiviral Rep-like sequences from eukaryotes, bacteria, plasmids, viruses and viral metagenomes.

The phylogenetic tree was built using PhyML-mixtures based on a multiple sequence alignment generated using COBALT with the Word Size parameter setting to 3. The base tree was drawn using Interactive Tree Of Life Version 1.9 (http://itol.embl.de/#). The protozoan sequences were chosen as outgroup for presentation purposes. The p-values of approximate likelihood ratios (SH-test) plotted as circle marks on the branches (only p-values >0.5 are indicated) and circle size is proportional to the p-values. Branch colors correspond to taxonomic groups and the main taxonomic groups are indicated. Scale bars correspond to 0.1 amino acid substitutions per site. Sequence accession numbers are given for each sequence.

Figure S6 Alignment of viral insertion loci in a genome.

For each species, a diagram of a representative viral insertion locus is shown. The related viral insertion loci from the same species were aligned with the representative viral insertion locus using BLASTn. Rectangular boxes with arrowheads indicate endogenous viral sequences: red, Rep-like sequences; blue, capsid-like sequences. The multi-color bar at the bottom indicates

the similarity levels as measured by BLAST scores.

Figure S7 Phylogeny of viral Rep-like sequences from parvovirus-like transposons with those of related extant viruses.

The phylogenetic tree was built using PhyML-mixtures based on a multiple sequence alignment generated using COBALT with the Constraint E-value parameter setting to 0.1. This tree was rooted with Rep_2 domain sequences from bacterial plasmid and bacterial genome. The topology of blue asterisk marked clade was evaluated independently. Only p-values of the approximate likelihood ratios (SH-test) >0.5(50%) are indicated. All scale bars correspond to 0.5 amino acid substitutions per site. Sequence accession numbers are given for each sequence.

Figure S8 Phylogenies of the full-length, the N-terminal and C-terminal regions of circoviral Rep-like proteins

The phylogenetic trees were built using PhyML-mixtures based on multiple sequence alignments generated using MCOFFEE. Only p-values of the approximate likelihood ratios (SH-test) >0.5(50%) are indicated. All scale bars correspond to 0.5 amino acid substitutions per site. Sequence accession numbers are given for each sequence. (A) Phylogenetic tree of N-terminal sequence. (B) Phylogenetic tree of C-terminal sequence. These two trees were rooted with Rep_2 domain sequences from bacterial plasmids and bacterial genomes. (C) Phylogenetic tree of full-length Rep sequences. The geminivirus-like sequences used as an outgroup base on tree (A) and (B). (D) Conserved motifs comparison of different Rep genes. The different Rep catalytic domain families and conserved motifs are indicated.

Figure S9 Phylogenies of the full-length, the N-terminal and C-terminal regions of geminiviral Rep-like proteins

The phylogenetic trees of full-length (A), N-terminal (B), C-terminal (C) sequences of representative geminivirus-like Rep were built respectively using PhyML-mixtures based on multiple sequence alignments generated using MCOFFEE. These three trees were rooted with nanovirus *Faba bean necrotic stunt virus* DNA R. Only p-values of the approximate likelihood ratios (SH-test) >0.5(50%) are indicated. All scale bars correspond to 0.5 amino acid substitutions per site. Sequence accession numbers are given for each sequence.

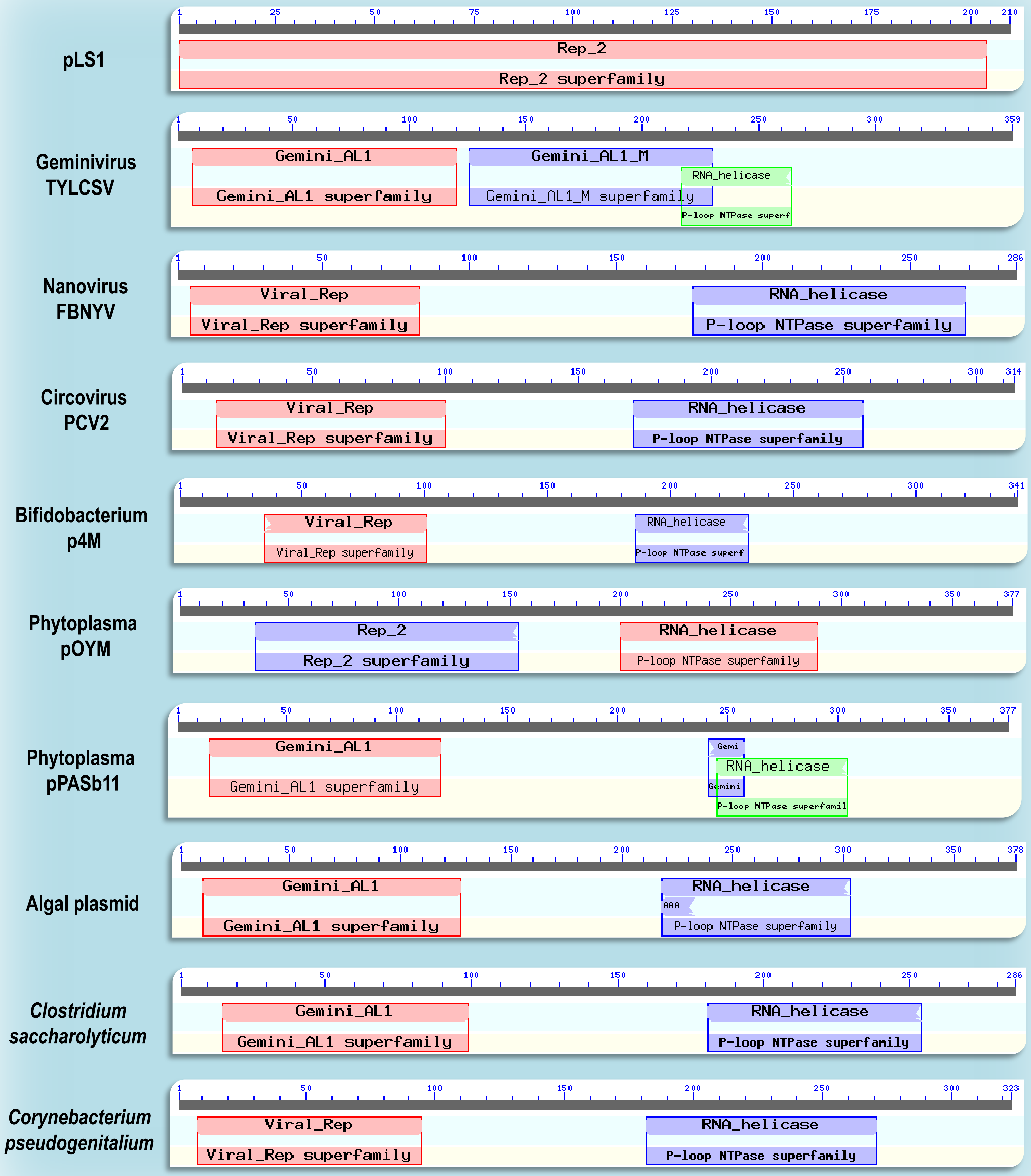
pLS1
Rep_2
Rep_2 superfamily
Geminivirus TYLCSV
Gemini_AL1
Gemini_AL1 superfamily
Gemini_AL1_M
Gemini_AL1_M superfamily
RNA_helicase
P-loop NTPase superf
Nanovirus FBNYV
Viral_Rep
Viral_Rep superfamily
RNA_helicase
P-loop NTPase superfamily
Circovirus PCV2
Viral_Rep
Viral_Rep superfamily
RNA_helicase
P-loop NTPase superfamily
Bifidobacterium p4M
Viral_Rep
Viral_Rep superfamily
RNA_helicase
P-loop NTPase superf
Phytoplasma pOYM
Rep_2
Rep_2 superfamily
RNA_helicase
P-loop NTPase superfamily
Phytoplasma pPASb11
Gemini_AL1
Gemini_AL1 superfamily
Gemi
Gemini
RNA_helicase
P-loop NTPase superfamil
Algal plasmid
Gemini_AL1
Gemini_AL1 superfamily
RNA_helicase
AAA
P-loop NTPase superfamily
Clostridium saccharolyticum
Gemini_AL1
Gemini_AL1 superfamily
RNA_helicase
P-loop NTPase superfamily
Corynebacterium pseudogenitalium
Viral_Rep
Viral_Rep superfamily
RNA_helicase
P-loop NTPase superfamily

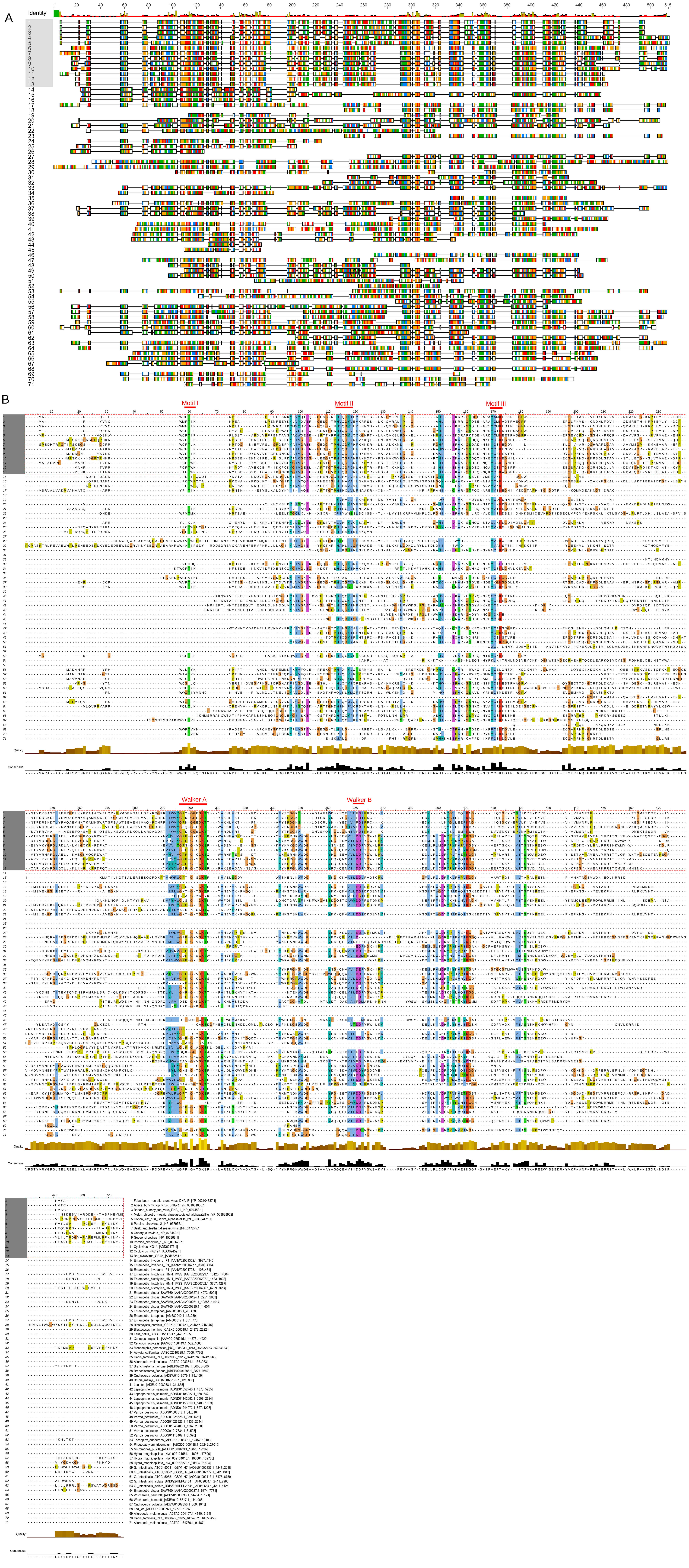
A
Identity
B
Motif I
Motif II
Motif III
Walker A
Walker B
Quality
Consensus
1 Faba_bean_necrotic_stunt_virus_DNA_R_|YP_003104737.1|
2 Abaca_bunchy_top_virus_DNA-R_|YP_001661660.1|
3 Banana_bunchy_top_virus_DNA_1_|NP_604483.1|
4 Melon_chlorotic_mosaic_virus-associated_alphasatellite_|YP_003828902|
5 Cotton_leaf_curl_Gezira_alphasatellite_|YP_003334471.1|
6 Porcine_circovirus_2_|NP_937956.1|
7 Beak_and_feather_disease_virus_|NP_047275.1|
8 Canary_circovirus_|NP_573442.1|
9 Goose_circovirus_|NP_150368.1|
10 Porcine_circovirus_1_|NP_065678.1|
11 Cyclovirus_NG14_|ADD62473.1|
12 Cyclovirus_PK6197_|ADD62459.1|
13 Bat_cyclovirus_GF-4c_|ADI48251.1|
14 Entamoeba_invadens_IP1_|AANW02001352.1_3997..4345|
15 Entamoeba_invadens_IP1_|AANW02001627.1_3316..4164|
16 Entamoeba_invadens_IP1_|AANW02004798.1_108..431|
17 Entamoeba_histolytica_HM-1_IMSS_|AAFB02000299.1_13120..14004|
18 Entamoeba_histolytica_HM-1_IMSS_|AAFB02000227.1_1483..1938|
19 Entamoeba_histolytica_HM-1_IMSS_|AAFB02000762.1_3767..4287|
20 Entamoeba_histolytica_HM-1_IMSS_|AAFB02000406.1_6739..7614|
21 Entamoeba_dispar_SAW760_|AANV02000527.1_4273..5091|
22 Entamoeba_dispar_SAW760_|AANV02000124.1_2251..2963|
23 Entamoeba_dispar_SAW760_|AANV02000261.1_10556..11017|
24 Entamoeba_dispar_SAW760_|AANV02000835.1_1..601|
25 Entamoeba_terrapinae_|AM688208.1_76..438|
26 Entamoeba_terrapinae_|AM680040.1_12..239|
27 Entamoeba_terrapinae_|AM666017.1_351..776|
28 Blastocystis_hominis_|CABX01000042.1_214657..216345|
29 Blastocystis_hominis_|CABX01000019.1_24873..26224|
30 Felis_catus_|ACBE01511791.1_443..1355|
31 Xenopus_tropicalis_|AAMC01055245.1_14573..14920|
32 Xenopus_tropicalis_|AAMC01188449.1_562..1080|
33 Monodelphis_domestica_|NC_008803.1_chr3_262232423..262233230|
34 Aplysia_californica_|AASC02010328.1_7506..7796|
35 Canis_familiaris_|NC_006599.2_chr17_37420760..37420963|
36 Ailuropoda_melanoleuca_|ACTA01008384.1_136..973|
37 Branchiostoma_floridae_|ABEP02021162.1_3600..4500|
38 Branchiostoma_floridae_|ABEP02001286.1_8877..9507|
39 Onchocerca_volvulus_|ADBW01019579.1_79..459|
40 Brugia_malayi_|AAQA01022198.1_121..800|
41 Loa_loa_|ADBU01008988.1_31..855|
42 Lepeophtheirus_salmonis_|ADND01052740.1_4873..5735|
43 Lepeophtheirus_salmonis_|ADND01186227.1_168..642|
44 Lepeophtheirus_salmonis_|ADND01142652.1_2508..2624|
45 Lepeophtheirus_salmonis_|ADND01156619.1_1403..1563|
46 Lepeophtheirus_salmonis_|ADND01244072.1_827..1203|
47 Varroa_destructor_|ADDG01008812.1_34..818|
48 Varroa_destructor_|ADDG01025628.1_959..1459|
49 Varroa_destructor_|ADDG01026923.1_1336..2044|
50 Varroa_destructor_|ADDG01043406.1_1367..2060|
51 Varroa_destructor_|ADDG01017834.1_6..503|
52 Varroa_destructor_|ADDG01113407.1_5..378|
53 Trichoplax_adhaerens_|ABGP01000147.1_12452..13183|
54 Phaeodactylum_tricornutum_|ABQD01000138.1_26242..27015|
55 Micromonas_pusilla_|ACCP01000489.1_18825..19202|
56 Hydra_magnipapillata_|NW_002121584.1_46961..47806|
57 Hydra_magnipapillata_|NW_002164010.1_108864..109788|
58 Hydra_magnipapillata_|NW_002153279.1_20604..21504|
59 G._intestinalis_ATCC_50581_GS/M_H7_|ACGJ01002837.1_1247..2218|
60 G._intestinalis_ATCC_50581_GS/M_H7_|ACGJ01002772.1_342..1343|
61 G._intestinalis_ATCC_50581_GS/M_H7_|ACGJ01002413.1_6178..6759|
62 G._intestinalis_isolate_BRIS/92/HEPU/1541_|AF059664.1_2411..2986|
63 G._intestinalis_isolate_BRIS/92/HEPU/1541_|AF059664.1_4211..5125|
64 Entamoeba_dispar_SAW760_|AANV02000527.1_6874..7771|
65 Wuchereria_bancrofti_|ADBV01000333.1_14404..15171|
66 Wuchereria_bancrofti_|ADBV01018817.1_144..968|
67 Onchocerca_volvulus_|ADBW01007856.1_669..1043|
68 Loa_loa_|ADBU01000376.1_12779..13360|
69 Ailuropoda_melanoleuca_|ACTA01004107.1_4780..5134|
70 Canis_familiaris_|NC_006604.2_chr22_64349520..64350453|
71 Ailuropoda_melanoleuca_|ACTA01184789.1_9..487|

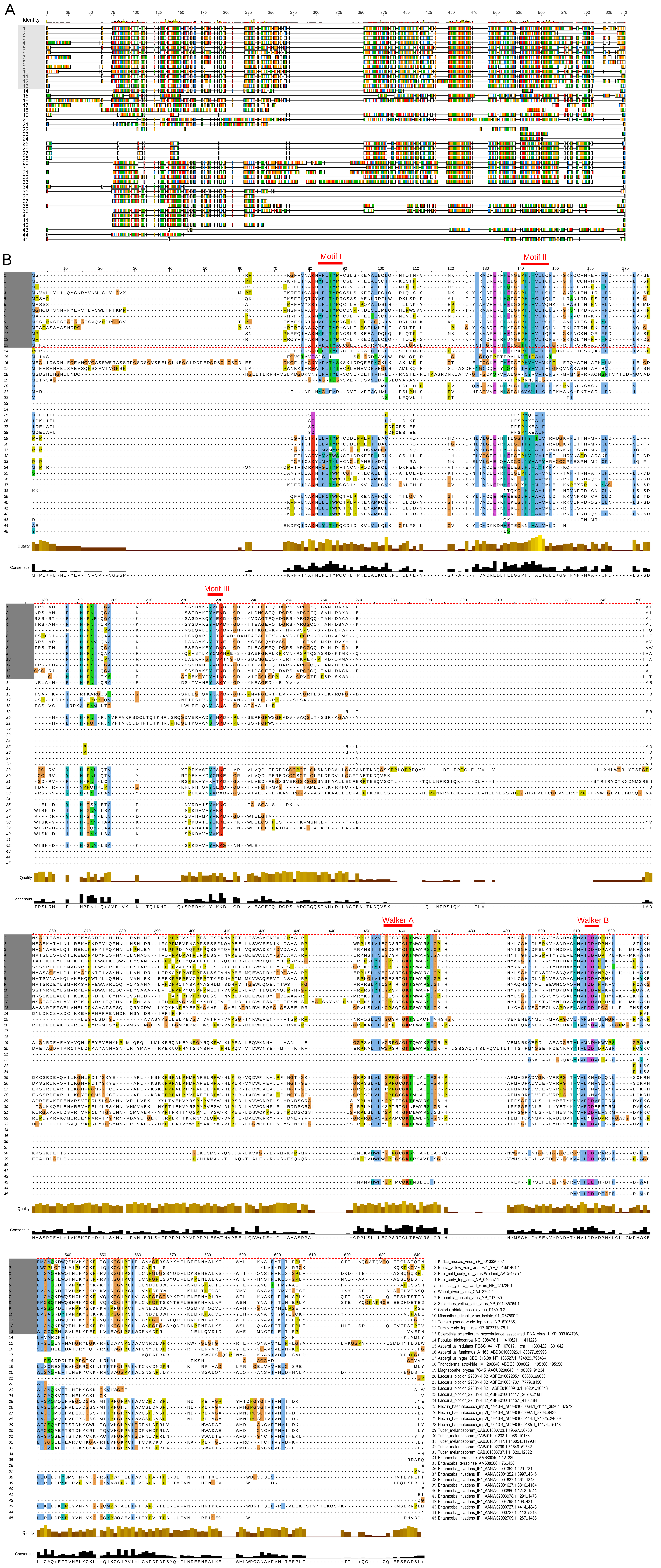
A
Identity
B
Motif I
Motif II
Motif III
Walker A
Walker B
Quality
Consensus
1 Kudzu_mosaic_virus_YP_001333680.1
2 Emilia_yellow_vein_virus-Fz1_YP_001661461.1
3 Beet_mild_curly_top_virus-Worland_AAC54875.1
4 Beet_curly_top_virus_NP_040557.1
5 Tobacco_yellow_dwarf_virus_NP_620726.1
6 Wheat_dwarf_virus_CAJ13704.1
7 Euphorbia_mosaic_virus_YP_717930.1
8 Spilanthes_yellow_vein_virus_YP_001285764.1
9 Chloris_striate_mosaic_virus_P18919.2
10 Miscanthus_streak_virus_isolate_91_Q67590.2
11 Tomato_pseudo-curly_top_virus_NP_620735.1
12 Turnip_curly_top_virus_YP_003778178.1
13 Sclerotinia_sclerotiorum_hypovirulence_associated_DNA_virus_1_YP_003104796.1
14 Populus_trichocarpa_NC_008478.1_11410621..11411228
15 Aspergillus_nidulans_FGSC_A4_NT_107012.1_chr_II_1300422..1301042
16 Aspergillus_fumigatus_A1163_ABDB01000026.1_88877..89998
17 Aspergillus_niger_CBS_513.88_NT_166527.1_794829..795464
18 Trichoderma_atroviride_IMI_206040_ABDG01000062.1_195366..195950
19 Magnaporthe_oryzae_70-15_AACU02000431.1_90509..91234
20 Laccaria_bicolor_S238N-H82_ABFE01002205.1_68683..69683
21 Laccaria_bicolor_S238N-H82_ABFE01000173.1_7779..8450
22 Laccaria_bicolor_S238N-H82__ABFE01000943.1_16201..16343
23 Laccaria_bicolor_S238N-H82_ABFE01001411.1_2070..2168
24 Laccaria_bicolor_S238N-H82_ABFE01001115.1_410..484
25 Nectria_haematococca_mpVI_77-13-4_ACJF01000064.1_chr14_36904..37572
26 Nectria_haematococca_mpVI_77-13-4_ACJF01000097.1_8768..9433
27 Nectria_haematococca_mpVI_77-13-4_ACJF01000114.1_24025..24699
28 Nectria_haematococca_mpVI_77-13-4_ACJF01000185.1_14474..15148
29 Tuber_melanosporum_CABJ01000723.1:49567..50703
30 Tuber_melanosporum_CABJ01001208.1:9066..10188
31 Tuber_melanosporum_CABJ01001447.1:116854..117984
32 Tuber_melanosporum_CABJ01002799.1:51549..52532
33 Tuber_melanosporum_CABJ01003737.1:11320..12522
34 Entamoeba_terrapinae_AM680040.1:12..239
35 Entamoeba_terrapinae_AM688208.1:76..438
36 Entamoeba_invadens_IP1_AANW02001352.1:429..731
37 Entamoeba_invadens_IP1_AANW02001352.1:3997..4345
38 Entamoeba_invadens_IP1_AANW02001627.1:561..1343
39 Entamoeba_invadens_IP1_AANW02001627.1:3316..4164
40 Entamoeba_invadens_IP1_AANW02003860.1:1242..1544
41 Entamoeba_invadens_IP1_AANW02003978.1:1291..1473
42 Entamoeba_invadens_IP1_AANW02004798.1:108..431
43 Entamoeba_invadens_IP1_AANW02000727.1:4414..4848
44 Entamoeba_invadens_IP1_AANW02000727.1:5113..5313
45 Entamoeba_invadens_IP1_AANW02002709.1:1267..1488

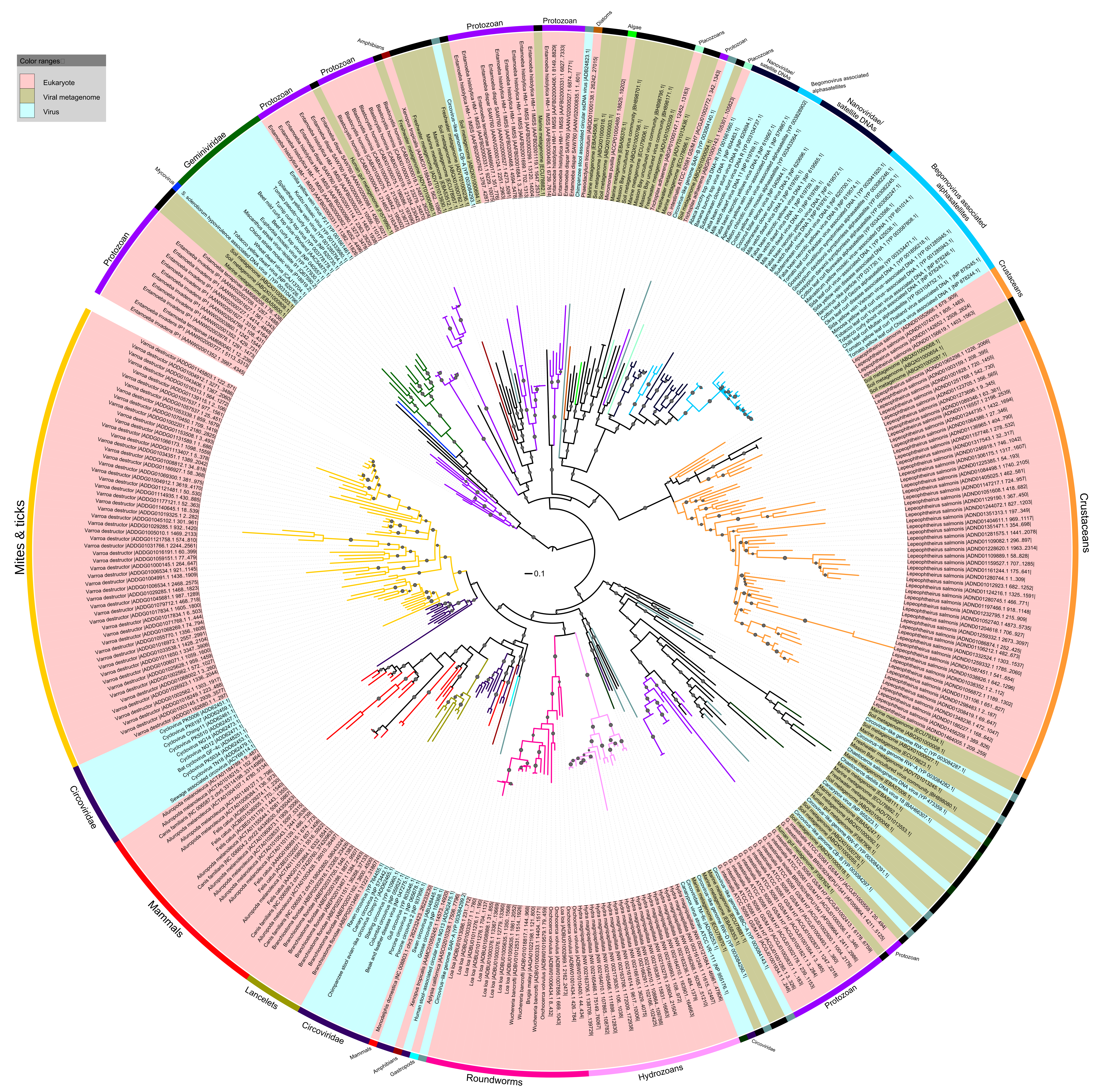

Color ranges
Eukaryote
Viral metagenome
Virus
Protozoan
Protozoan
Diatoms
Algae
Placozoans
Protozoan
Placozoans
Amphibians
Protozoan
Protozoan
Geminiviridae
Mycovirus
Protozoan
Nanoviridae/ satellite DNAs
Begomovirus associated alphasatellites
Nanoviridae/ satellite DNAs
Begomovirus associated alphasatellites
Crustaceans
Crustaceans
Mites & ticks
Circoviridae
Mammals
Lancelets
Circoviridae
Mammals
Amphibians
Gastropods
Roundworms
Hydrozoans
Circoviridae
Protozoan
Protozoan
0.1

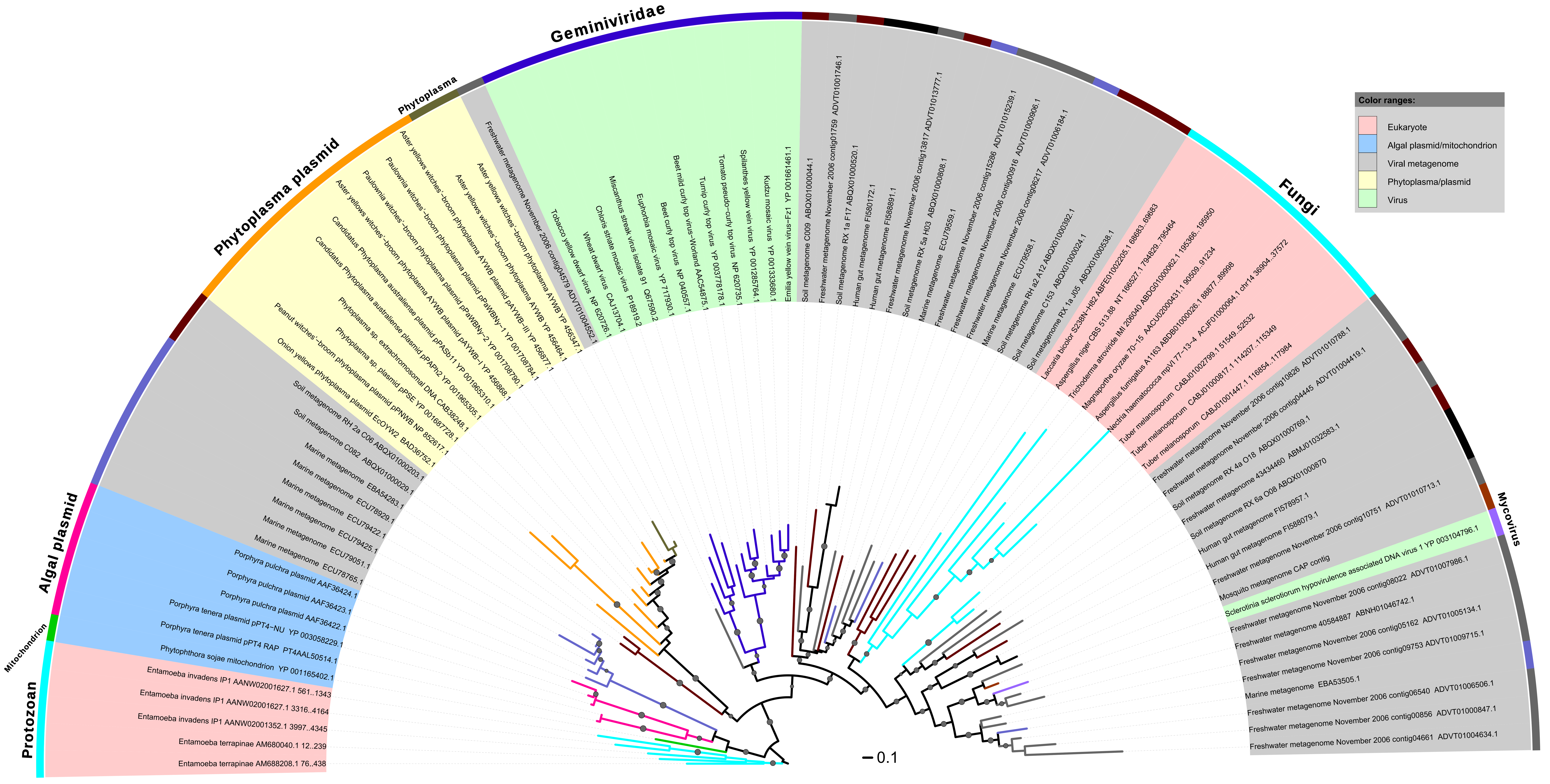

Color ranges:
Eukaryote
Algal plasmid/mitochondrion
Viral metagenome
Phytoplasma/plasmid
Virus
Protozoan
Mitochondrion
Algal plasmid
Phytoplasma plasmid
Phytoplasma
Geminiviridae
Fungi
Mycovirus
Entamoeba terrapinae AM688208.1 76..438
Entamoeba terrapinae AM680040.1 12..239
Entamoeba invadens IP1 AANW02001352.1 3997..4345
Entamoeba invadens IP1 AANW02001627.1 3316..4164
Entamoeba invadens IP1 AANW02001627.1 561..1343
Phytophthora sojae mitochondrion YP 001165402.1
Porphyra tenera plasmid pPT4 RAP PT4AAL50514.1
Porphyra tenera plasmid pPT4−NU YP 003058229.1
Porphyra pulchra plasmid AAF36422.1
Porphyra pulchra plasmid AAF36423.1
Porphyra pulchra plasmid AAF36424.1
Marine metagenome ECU78765.1
Marine metagenome ECU79051.1
Marine metagenome ECU79425.1
Marine metagenome ECU79422.1
Marine metagenome ECU78929.1
Marine metagenome EBA54283.1
Soil metagenome C082 ABQX01000029.1
Soil metagenome RH 2a C06 ABQX01000203.1
Onion yellows phytoplasma plasmid EcOYW2 BAD36752.1
Peanut witches−broom phytoplasma plasmid pPNWB NP 852617.1
Phytoplasma sp. plasmid pPSE YP 001687728.1
Phytoplasma sp. extrachromosomal DNA CAB38248.1
Candidatus Phytoplasma australiense plasmid pPAPh2 YP 001965305.1
Candidatus Phytoplasma australiense plasmid pPASb11 YP 001965310.1
Aster yellows witches−broom phytoplasma AYWB plasmid pAYWB−I YP 456868.1
Paulownia witches−broom phytoplasma plasmid pPaWBNy−2 YP 001708790.1
Paulownia witches−broom phytoplasma plasmid pPaWBNy−1 YP 001708784.1
Aster yellows witches−broom phytoplasma AYWB plasmid pAYWB−III YP 456877.1
Aster yellows witches−broom phytoplasma AYWB YP 456464.1
Aster yellows witches−broom phytoplasma AYWB YP 456347.1
Freshwater metagenome November 2006 contig04579 ADVT01004552.1
Tobacco yellow dwarf virus NP 620726.1
Wheat dwarf virus CAJ13704.1
Chloris striate mosaic virus P18919.2
Miscanthus streak virus isolate 91 Q67590.2
Euphorbia mosaic virus YP 717930.1
Beet curly top virus NP 040557.1
Beet mild curly top virus−Worland AAC54875.1
Turnip curly top virus YP 003778178.1
Tomato pseudo−curly top virus NP 620735.1
Spilanthes yellow vein virus YP 001285764.1
Kudzu mosaic virus YP 001333680.1
Emilia yellow vein virus−Fz1 YP 001661461.1
Soil metagenome C009 ABQX01000044.1
Freshwater metagenome November 2006 contig01759 ADVT01001746.1
Soil metagenome RX 1a F17 ABQX01000520.1
Human gut metagenome FI580172.1
Human gut metagenome FI588891.1
Freshwater metagenome November 2006 contig13817 ADVT01013777.1
Soil metagenome RX 5a H03 ABQX01000808.1
Marine metagenome ECU79559.1
Freshwater metagenome November 2006 contig15286 ADVT01015239.1
Freshwater metagenome November 2006 contig00916 ADVT01000906.1
Freshwater metagenome November 2006 contig06217 ADVT01006184.1
Marine metagenome ECU79558.1
Soil metagenome RH a2 A12 ABQX01000392.1
Soil metagenome C153 ABQX01000024.1
Soil metagenome RX 1a J05 ABQX01000538.1
Laccaria bicolor S238N−H82 ABFE01002205.1 68683..69683
Aspergillus niger CBS 513.88 NT 166527.1 794829..795464
Trichoderma atroviride IMI 206040 ABDG01000062.1 195366..195950
Magnaporthe oryzae 70−15 AACU02000431.1 90509..91234
Aspergillus fumigatus A1163 ABDB01000026.1 88877..89998
Nectria haematococca mpVI 77−13−4 ACJF01000064.1 chr14 36904..37572
Tuber melanosporum CABJ01002799.1 51549..52532
Tuber melanosporum CABJ01000817.1 114207..115349
Tuber melanosporum CABJ01001447.1 116854..117984
Freshwater metagenome November 2006 contig10826 ADVT01010788.1
Freshwater metagenome November 2006 contig04445 ADVT01004419.1
Soil metagenome RX 4a O18 ABQX01000769.1
Freshwater metagenome 43434460 ABMJ01032583.1
Soil metagenome RX 6a O08 ABQX01000870
Human gut metagenome FI578957.1
Human gut metagenome FI588079.1
Freshwater metagenome November 2006 contig10751 ADVT01010713.1
Mosquito metagenome CAP contig
Sclerotinia sclerotiorum hypovirulence associated DNA virus 1 YP 003104796.1
Freshwater metagenome November 2006 contig08022 ADVT01007986.1
Freshwater metagenome 40584887 ABNH01046742.1
Freshwater metagenome November 2006 contig05162 ADVT01005134.1
Freshwater metagenome November 2006 contig09753 ADVT01009715.1
Marine metagenome EBA53505.1
Freshwater metagenome November 2006 contig06540 ADVT01006506.1
Freshwater metagenome November 2006 contig00856 ADVT01000847.1
Freshwater metagenome November 2006 contig04661 ADVT01004634.1
0.1

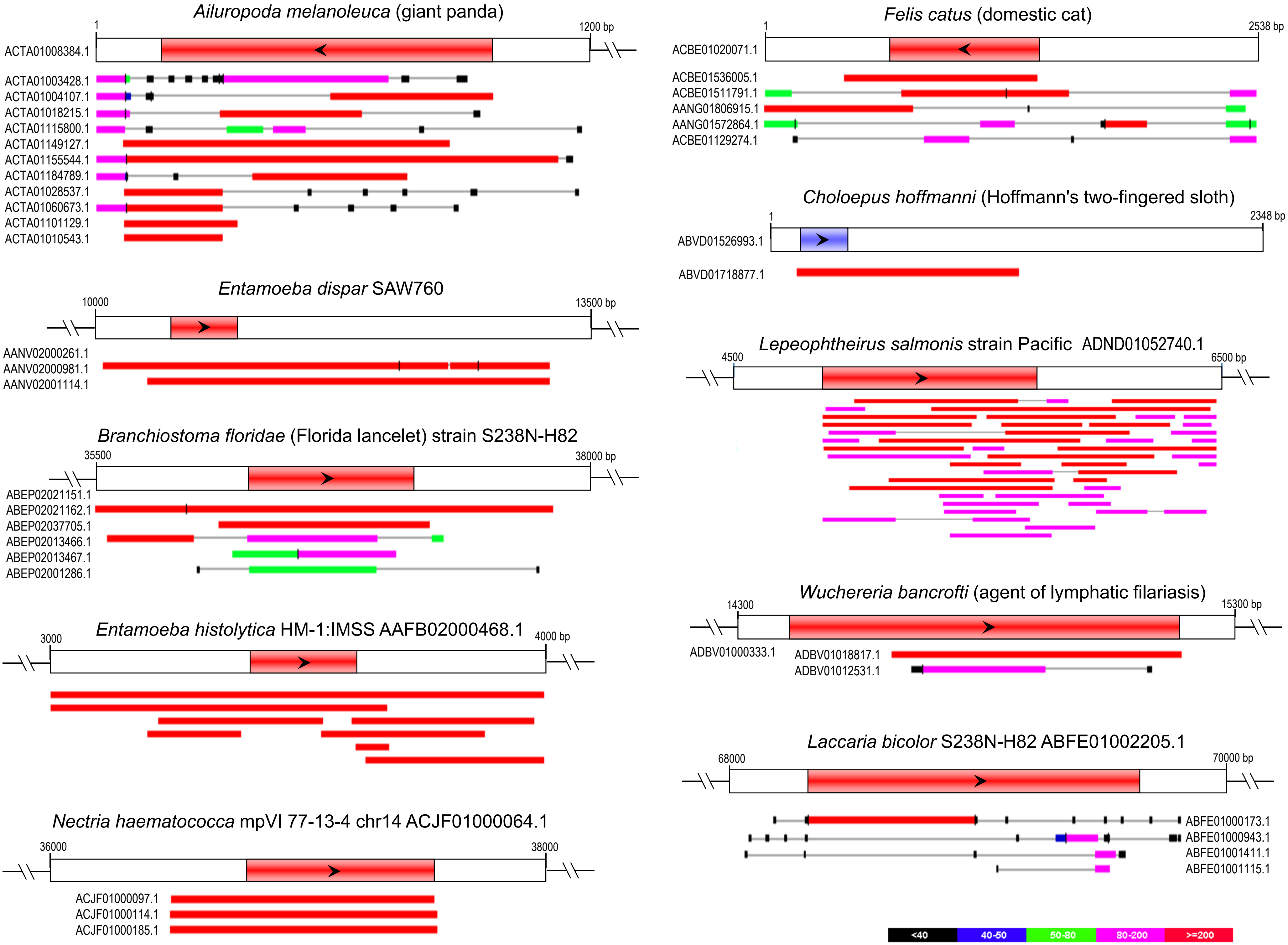

Ailuropoda melanoleuca (giant panda)
1
1200 bp
ACTA01008384.1
ACTA01003428.1
ACTA01004107.1
ACTA01018215.1
ACTA01115800.1
ACTA01149127.1
ACTA01155544.1
ACTA01184789.1
ACTA01028537.1
ACTA01060673.1
ACTA01101129.1
ACTA01010543.1
Felis catus (domestic cat)
1
2538 bp
ACBE01020071.1
ACBE01536005.1
ACBE01511791.1
AANG01806915.1
AANG01572864.1
ACBE01129274.1
Choloepus hoffmanni (Hoffmann's two-fingered sloth)
1
2348 bp
ABVD01526993.1
ABVD01718877.1
Entamoeba dispar SAW760
10000
13500 bp
AANV02000261.1
AANV02000981.1
AANV02001114.1
Lepeophtheirus salmonis strain Pacific ADND01052740.1
4500
6500 bp
Branchiostoma floridae (Florida lancelet) strain S238N-H82
35500
38000 bp
ABEP02021151.1
ABEP02021162.1
ABEP02037705.1
ABEP02013466.1
ABEP02013467.1
ABEP02001286.1
Wuchereria bancrofti (agent of lymphatic filariasis)
14300
15300 bp
ADBV01000333.1
ADBV01018817.1
ADBV01012531.1
Entamoeba histolytica HM-1:IMSS AAFB02000468.1
3000
4000 bp
Laccaria bicolor S238N-H82 ABFE01002205.1
68000
70000 bp
ABFE01000173.1
ABFE01000943.1
ABFE01001411.1
ABFE01001115.1
Nectria haematococca mpVI 77-13-4 chr14 ACJF01000064.1
36000
38000
ACJF01000097.1
ACJF01000114.1
ACJF01000185.1
<40
40-50
50-80
80-200
>=200

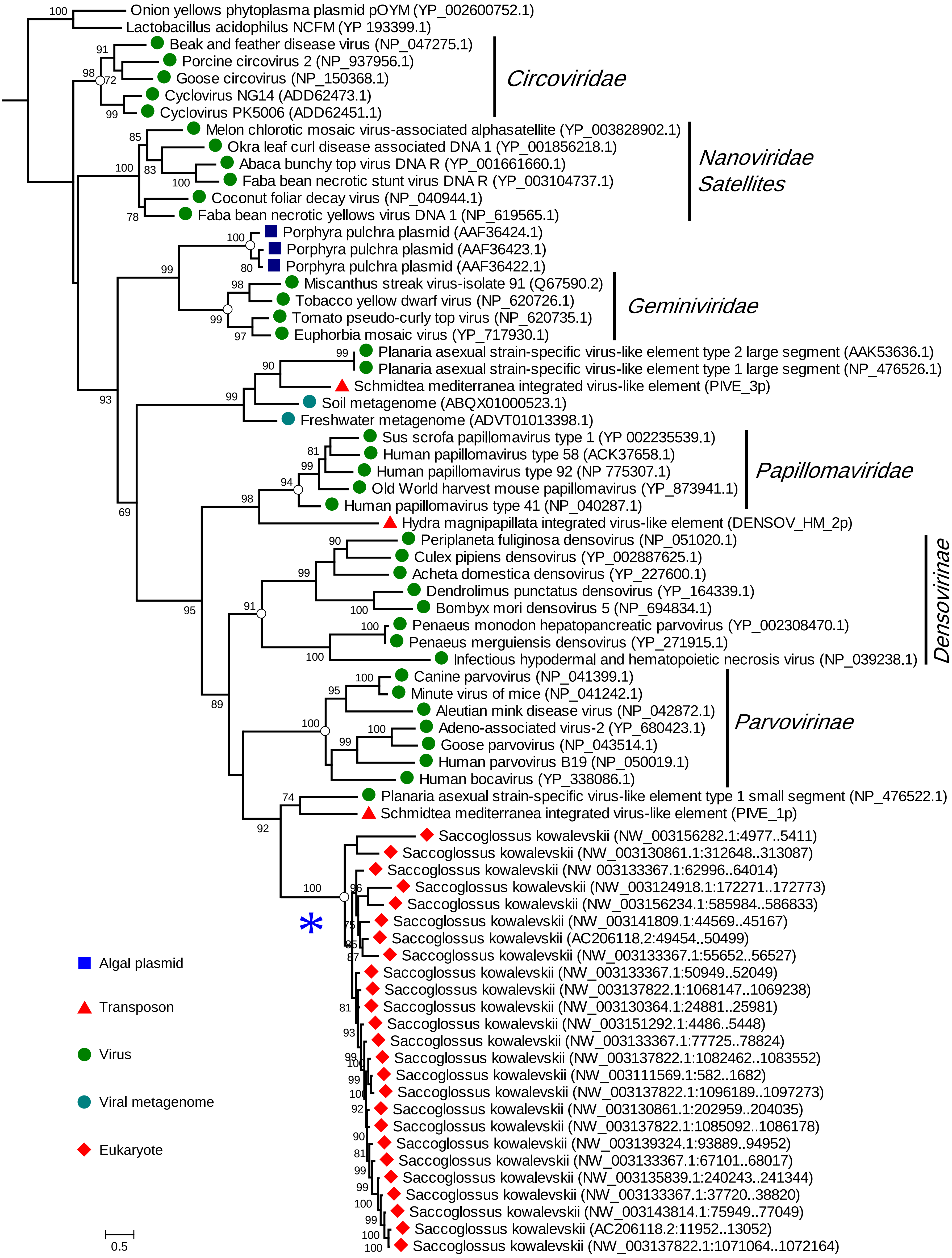

Onion yellows phytoplasma plasmid pOYM (YP_002600752.1)
Lactobacillus acidophilus NCFM (YP 193399.1)
Beak and feather disease virus (NP_047275.1)
Porcine circovirus 2 (NP_937956.1)
Goose circovirus (NP_150368.1)
Cyclovirus NG14 (ADD62473.1)
Cyclovirus PK5006 (ADD62451.1)
Circoviridae
Melon chlorotic mosaic virus-associated alphasatellite (YP_003828902.1)
Okra leaf curl disease associated DNA 1 (YP_001856218.1)
Abaca bunchy top virus DNA R (YP_001661660.1)
Faba bean necrotic stunt virus DNA R (YP_003104737.1)
Coconut foliar decay virus (NP_040944.1)
Faba bean necrotic yellows virus DNA 1 (NP_619565.1)
Nanoviridae
Satellites
Porphyra pulchra plasmid (AAF36424.1)
Porphyra pulchra plasmid (AAF36423.1)
Porphyra pulchra plasmid (AAF36422.1)
Miscanthus streak virus-isolate 91 (Q67590.2)
Tobacco yellow dwarf virus (NP_620726.1)
Tomato pseudo-curly top virus (NP_620735.1)
Euphorbia mosaic virus (YP_717930.1)
Geminiviridae
Planaria asexual strain-specific virus-like element type 2 large segment (AAK53636.1)
Planaria asexual strain-specific virus-like element type 1 large segment (NP_476526.1)
Schmidtea mediterranea integrated virus-like element (PIVE_3p)
Soil metagenome (ABQX01000523.1)
Freshwater metagenome (ADVT01013398.1)
Sus scrofa papillomavirus type 1 (YP 002235539.1)
Human papillomavirus type 58 (ACK37658.1)
Human papillomavirus type 92 (NP 775307.1)
Old World harvest mouse papillomavirus (YP_873941.1)
Human papillomavirus type 41 (NP_040287.1)
Papillomaviridae
Hydra magnipapillata integrated virus-like element (DENSOV_HM_2p)
Periplaneta fuliginosa densovirus (NP_051020.1)
Culex pipiens densovirus (YP_002887625.1)
Acheta domestica densovirus (YP_227600.1)
Dendrolimus punctatus densovirus (YP_164339.1)
Bombyx mori densovirus 5 (NP_694834.1)
Penaeus monodon hepatopancreatic parvovirus (YP_002308470.1)
Penaeus merguiensis densovirus (YP_271915.1)
Infectious hypodermal and hematopoietic necrosis virus (NP_039238.1)
Densovirinae
Canine parvovirus (NP_041399.1)
Minute virus of mice (NP_041242.1)
Aleutian mink disease virus (NP_042872.1)
Adeno-associated virus-2 (YP_680423.1)
Goose parvovirus (NP_043514.1)
Human parvovirus B19 (NP_050019.1)
Human bocavirus (YP_338086.1)
Parvovirinae
Planaria asexual strain-specific virus-like element type 1 small segment (NP_476522.1)
Schmidtea mediterranea integrated virus-like element (PIVE_1p)
Saccoglossus kowalevskii (NW_003156282.1:4977..5411)
Saccoglossus kowalevskii (NW_003130861.1:312648..313087)
Saccoglossus kowalevskii (NW 003133367.1:62996..64014)
Saccoglossus kowalevskii (NW_003124918.1:172271..172773)
Saccoglossus kowalevskii (NW_003156234.1:585984..586833)
Saccoglossus kowalevskii (NW_003141809.1:44569..45167)
Saccoglossus kowalevskii (AC206118.2:49454..50499)
Saccoglossus kowalevskii (NW_003133367.1:55652..56527)
Saccoglossus kowalevskii (NW_003133367.1:50949..52049)
Saccoglossus kowalevskii (NW_003137822.1:1068147..1069238)
Saccoglossus kowalevskii (NW_003130364.1:24881..25981)
Saccoglossus kowalevskii (NW_003151292.1:4486..5448)
Saccoglossus kowalevskii (NW_003133367.1:77725..78824)
Saccoglossus kowalevskii (NW_003137822.1:1082462..1083552)
Saccoglossus kowalevskii (NW_003111569.1:582..1682)
Saccoglossus kowalevskii (NW_003137822.1:1096189..1097273)
Saccoglossus kowalevskii (NW_003130861.1:202959..204035)
Saccoglossus kowalevskii (NW_003137822.1:1085092..1086178)
Saccoglossus kowalevskii (NW_003139324.1:93889..94952)
Saccoglossus kowalevskii (NW_003133367.1:67101..68017)
Saccoglossus kowalevskii (NW_003135839.1:240243..241344)
Saccoglossus kowalevskii (NW_003133367.1:37720..38820)
Saccoglossus kowalevskii (NW_003143814.1:75949..77049)
Saccoglossus kowalevskii (AC206118.2:11952..13052)
Saccoglossus kowalevskii (NW_003137822.1:1071064..1072164)
*
Algal plasmid
Transposon
Virus
Viral metagenome
Eukaryote
0.5

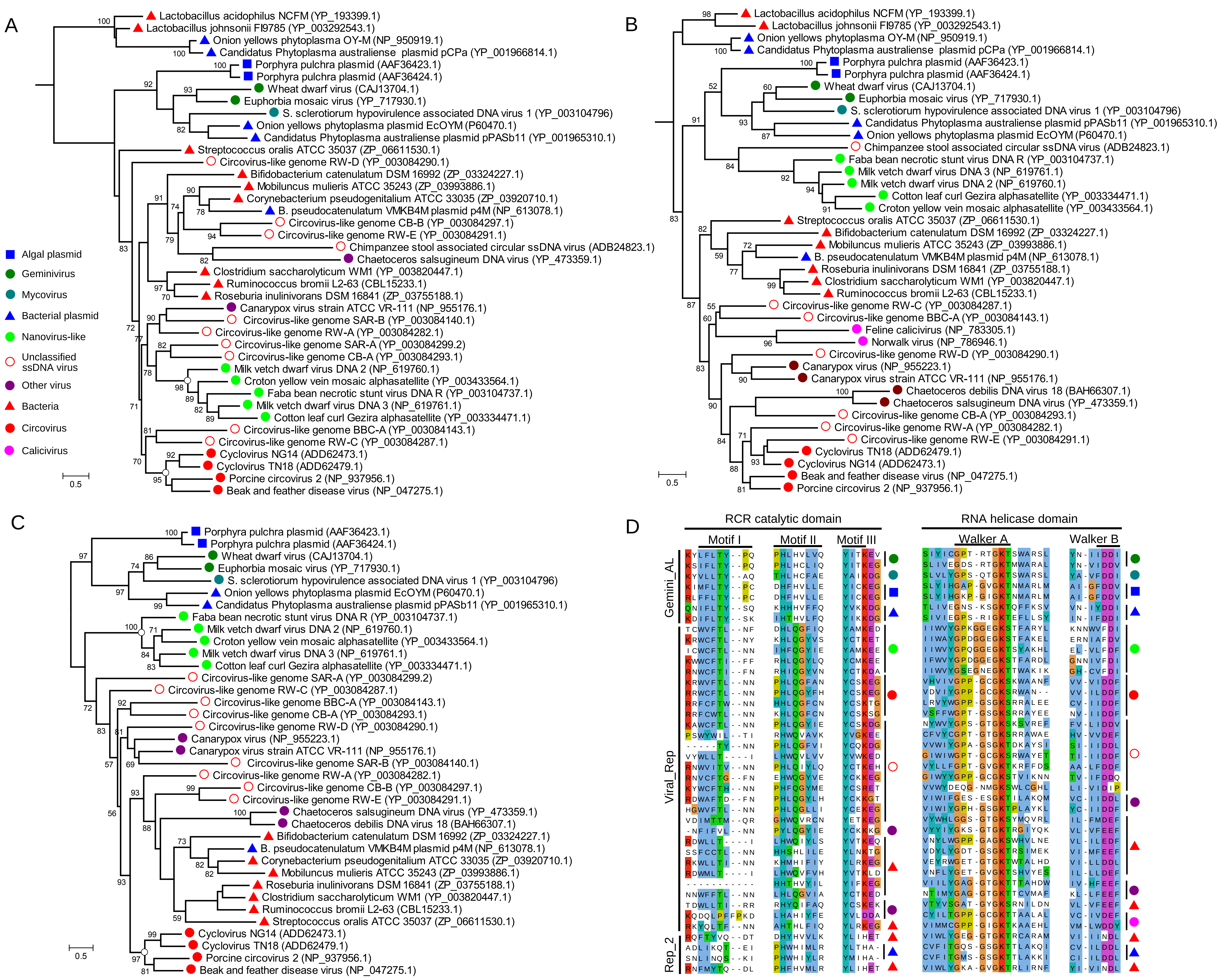

A
Lactobacillus acidophilus NCFM (YP_193399.1)
Lactobacillus johnsonii FI9785 (YP_003292543.1)
Onion yellows phytoplasma OY-M (NP_950919.1)
Candidatus Phytoplasma australiense plasmid pCPa (YP_001966814.1)
Porphyra pulchra plasmid (AAF36423.1)
Porphyra pulchra plasmid (AAF36424.1)
Wheat dwarf virus (CAJ13704.1)
Euphorbia mosaic virus (YP_717930.1)
S. sclerotiorum hypovirulence associated DNA virus 1 (YP_003104796)
Onion yellows phytoplasma plasmid EcOYM (P60470.1)
Candidatus Phytoplasma australiense plasmid pPASb11 (YP_001965310.1)
Streptococcus oralis ATCC 35037 (ZP_06611530.1)
Circovirus-like genome RW-D (YP_003084290.1)
Bifidobacterium catenulatum DSM 16992 (ZP_03324227.1)
Mobiluncus mulieris ATCC 35243 (ZP_03993886.1)
Corynebacterium pseudogenitalium ATCC 33035 (ZP_03920710.1)
B. pseudocatenulatum VMKB4M plasmid p4M (NP_613078.1)
Circovirus-like genome CB-B (YP_003084297.1)
Circovirus-like genome RW-E (YP_003084291.1)
Chimpanzee stool associated circular ssDNA virus (ADB24823.1)
Chaetoceros salsugineum DNA virus (YP_473359.1)
Clostridium saccharolyticum WM1 (YP_003820447.1)
Ruminococcus bromii L2-63 (CBL15233.1)
Roseburia inulinivorans DSM 16841 (ZP_03755188.1)
Canarypox virus strain ATCC VR-111 (NP_955176.1)
Circovirus-like genome SAR-B (YP_003084140.1)
Circovirus-like genome RW-A (YP_003084282.1)
Circovirus-like genome SAR-A (YP_003084299.2)
Circovirus-like genome CB-A (YP_003084293.1)
Milk vetch dwarf virus DNA 2 (NP_619760.1)
Croton yellow vein mosaic alphasatellite (YP_003433564.1)
Faba bean necrotic stunt virus DNA R (YP_003104737.1)
Milk vetch dwarf virus DNA 3 (NP_619761.1)
Cotton leaf curl Gezira alphasatellite (YP_003334471.1)
Circovirus-like genome BBC-A (YP_003084143.1)
Circovirus-like genome RW-C (YP_003084287.1)
Cyclovirus NG14 (ADD62473.1)
Cyclovirus TN18 (ADD62479.1)
Porcine circovirus 2 (NP_937956.1)
Beak and feather disease virus (NP_047275.1)
0.5
Algal plasmid
Geminivirus
Mycovirus
Bacterial plasmid
Nanovirus-like
Unclassified ssDNA virus
Other virus
Bacteria
Circovirus
Calicivirus
B
Lactobacillus acidophilus NCFM (YP_193399.1)
Lactobacillus johnsonii FI9785 (YP_003292543.1)
Onion yellows phytoplasma OY-M (NP_950919.1)
Candidatus Phytoplasma australiense plasmid pCPa (YP_001966814.1)
Porphyra pulchra plasmid (AAF36423.1)
Porphyra pulchra plasmid (AAF36424.1)
Wheat dwarf virus (CAJ13704.1)
Euphorbia mosaic virus (YP_717930.1)
S. sclerotiorum hypovirulence associated DNA virus 1 (YP_003104796)
Candidatus Phytoplasma australiense plasmid pPASb11 (YP_001965310.1)
Onion yellows phytoplasma plasmid EcOYM (P60470.1)
Chimpanzee stool associated circular ssDNA virus (ADB24823.1)
Faba bean necrotic stunt virus DNA R (YP_003104737.1)
Milk vetch dwarf virus DNA 3 (NP_619761.1)
Milk vetch dwarf virus DNA 2 (NP_619760.1)
Cotton leaf curl Gezira alphasatellite (YP_003334471.1)
Croton yellow vein mosaic alphasatellite (YP_003433564.1)
Streptococcus oralis ATCC 35037 (ZP_06611530.1)
Bifidobacterium catenulatum DSM 16992 (ZP_03324227.1)
Mobiluncus mulieris ATCC 35243 (ZP_03993886.1)
B. pseudocatenulatum VMKB4M plasmid p4M (NP_613078.1)
Roseburia inulinivorans DSM 16841 (ZP_03755188.1)
Clostridium saccharolyticum WM1 (YP_003820447.1)
Ruminococcus bromii L2-63 (CBL15233.1)
Circovirus-like genome RW-C (YP_003084287.1)
Circovirus-like genome BBC-A (YP_003084143.1)
Feline calicivirus (NP_783305.1)
Norwalk virus (NP_786946.1)
Circovirus-like genome RW-D (YP_003084290.1)
Canarypox virus (NP_955223.1)
Canarypox virus strain ATCC VR-111 (NP_955176.1)
Chaetoceros debilis DNA virus 18 (BAH66307.1)
Chaetoceros salsugineum DNA virus (YP_473359.1)
Circovirus-like genome CB-A (YP_003084293.1)
Circovirus-like genome RW-A (YP_003084282.1)
Circovirus-like genome RW-E (YP_003084291.1)
Cyclovirus TN18 (ADD62479.1)
Cyclovirus NG14 (ADD62473.1)
Beak and feather disease virus (NP_047275.1)
Porcine circovirus 2 (NP_937956.1)
0.5
C
Porphyra pulchra plasmid (AAF36423.1)
Porphyra pulchra plasmid (AAF36424.1)
Wheat dwarf virus (CAJ13704.1)
Euphorbia mosaic virus (YP_717930.1)
S. sclerotiorum hypovirulence associated DNA virus 1 (YP_003104796)
Onion yellows phytoplasma plasmid EcOYM (P60470.1)
Candidatus Phytoplasma australiense plasmid pPASb11 (YP_001965310.1)
Faba bean necrotic stunt virus DNA R (YP_003104737.1)
Milk vetch dwarf virus DNA 2 (NP_619760.1)
Croton yellow vein mosaic alphasatellite (YP_003433564.1)
Milk vetch dwarf virus DNA 3 (NP_619761.1)
Cotton leaf curl Gezira alphasatellite (YP_003334471.1)
Circovirus-like genome SAR-A (YP_003084299.2)
Circovirus-like genome RW-C (YP_003084287.1)
Circovirus-like genome BBC-A (YP_003084143.1)
Circovirus-like genome CB-A (YP_003084293.1)
Circovirus-like genome RW-D (YP_003084290.1)
Canarypox virus (NP_955223.1)
Canarypox virus strain ATCC VR-111 (NP_955176.1)
Circovirus-like genome SAR-B (YP_003084140.1)
Circovirus-like genome RW-A (YP_003084282.1)
Circovirus-like genome CB-B (YP_003084297.1)
Circovirus-like genome RW-E (YP_003084291.1)
Chaetoceros salsugineum DNA virus (YP_473359.1)
Chaetoceros debilis DNA virus 18 (BAH66307.1)
Bifidobacterium catenulatum DSM 16992 (ZP_03324227.1)
B. pseudocatenulatum VMKB4M plasmid p4M (NP_613078.1)
Corynebacterium pseudogenitalium ATCC 33035 (ZP_03920710.1)
Mobiluncus mulieris ATCC 35243 (ZP_03993886.1)
Roseburia inulinivorans DSM 16841 (ZP_03755188.1)
Clostridium saccharolyticum WM1 (YP_003820447.1)
Ruminococcus bromii L2-63 (CBL15233.1)
Streptococcus oralis ATCC 35037 (ZP_06611530.1)
Cyclovirus NG14 (ADD62473.1)
Cyclovirus TN18 (ADD62479.1)
Porcine circovirus 2 (NP_937956.1)
Beak and feather disease virus (NP_047275.1)
0.5
D
RCR catalytic domain
Motif I
Motif II
Motif III
RNA helicase domain
Walker A
Walker B
Gemini_AL
Viral_Rep
Rep_2

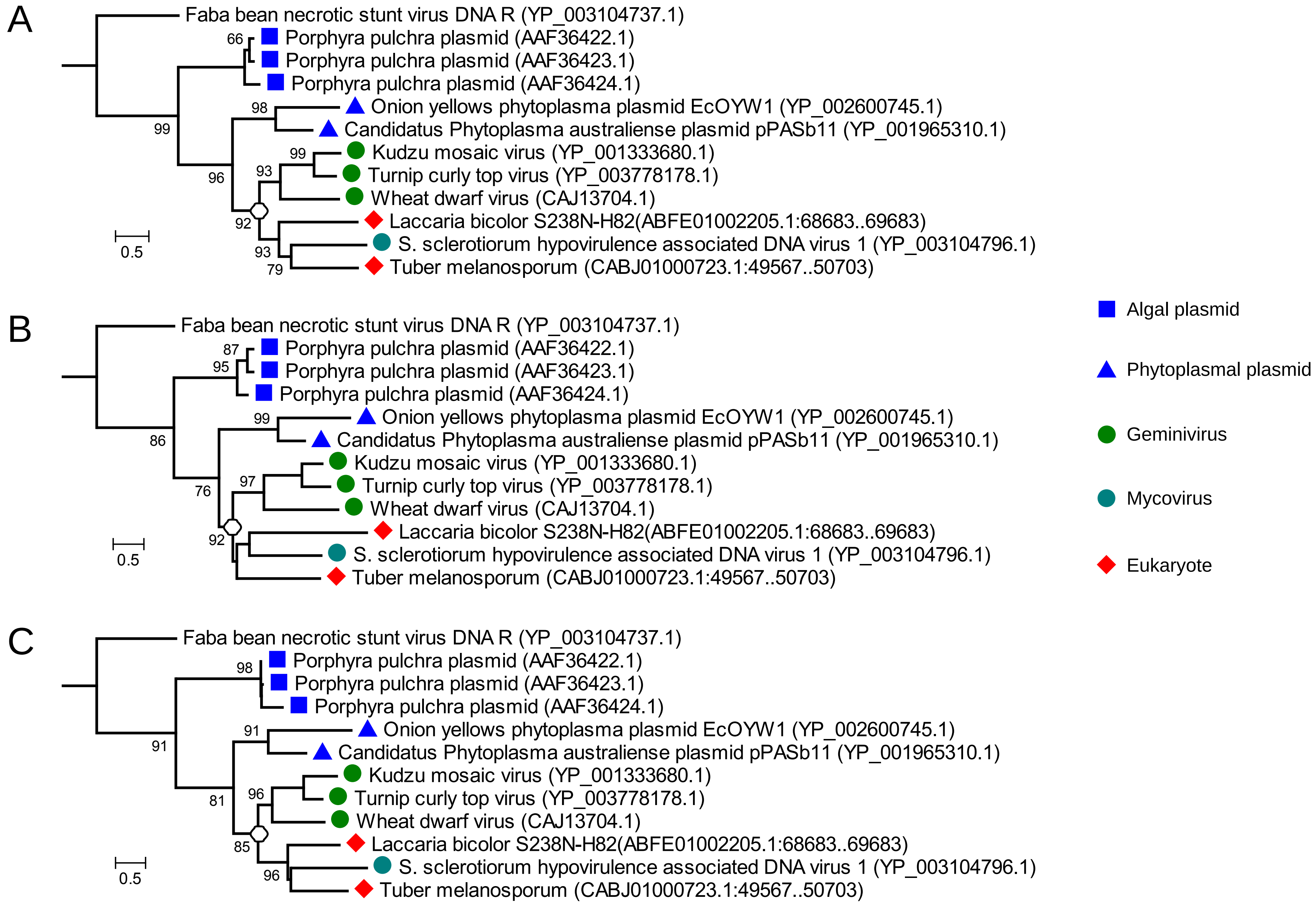

A
Faba bean necrotic stunt virus DNA R (YP_003104737.1)
66
Porphyra pulchra plasmid (AAF36422.1)
Porphyra pulchra plasmid (AAF36423.1)
Porphyra pulchra plasmid (AAF36424.1)
98
Onion yellows phytoplasma plasmid EcOYW1 (YP_002600745.1)
99
Candidatus Phytoplasma australiense plasmid pPASb11 (YP_001965310.1)
99
Kudzu mosaic virus (YP_001333680.1)
96
93
Turnip curly top virus (YP_003778178.1)
Wheat dwarf virus (CAJ13704.1)
92
Laccaria bicolor S238N-H82(ABFE01002205.1:68683..69683)
0.5
93
S. sclerotiorum hypovirulence associated DNA virus 1 (YP_003104796.1)
79
Tuber melanosporum (CABJ01000723.1:49567..50703)
B
Faba bean necrotic stunt virus DNA R (YP_003104737.1)
87
Porphyra pulchra plasmid (AAF36422.1)
95
Porphyra pulchra plasmid (AAF36423.1)
Porphyra pulchra plasmid (AAF36424.1)
99
Onion yellows phytoplasma plasmid EcOYW1 (YP_002600745.1)
86
Candidatus Phytoplasma australiense plasmid pPASb11 (YP_001965310.1)
Kudzu mosaic virus (YP_001333680.1)
76
97
Turnip curly top virus (YP_003778178.1)
Wheat dwarf virus (CAJ13704.1)
92
Laccaria bicolor S238N-H82(ABFE01002205.1:68683..69683)
0.5
S. sclerotiorum hypovirulence associated DNA virus 1 (YP_003104796.1)
Tuber melanosporum (CABJ01000723.1:49567..50703)
C
Faba bean necrotic stunt virus DNA R (YP_003104737.1)
98
Porphyra pulchra plasmid (AAF36422.1)
Porphyra pulchra plasmid (AAF36423.1)
Porphyra pulchra plasmid (AAF36424.1)
91
Onion yellows phytoplasma plasmid EcOYW1 (YP_002600745.1)
91
Candidatus Phytoplasma australiense plasmid pPASb11 (YP_001965310.1)
Kudzu mosaic virus (YP_001333680.1)
81
96
Turnip curly top virus (YP_003778178.1)
Wheat dwarf virus (CAJ13704.1)
85
Laccaria bicolor S238N-H82(ABFE01002205.1:68683..69683)
0.5
96
S. sclerotiorum hypovirulence associated DNA virus 1 (YP_003104796.1)
Tuber melanosporum (CABJ01000723.1:49567..50703)
Algal plasmid
Phytoplasmal plasmid
Geminivirus
Mycovirus
Eukaryote
