# Supplementary material for: Widespread Horizontal Gene Transfer from Circular Single-stranded DNA Viruses to Eukaryotic Genomes
Source: BMC Evol Biol. 2011 Sep 26;11:276. doi: 10.1186/1471-2148-11-276 (PMC3198968; doi:10.1186/1471-2148-11-276)
Supplement: Additional file 3 — supplementary table S1. This file lists primers used for PCR of endogenous virus-like regions of dog and cat genomes. [file 1471-2148-11-276-S3.PDF]

Table S1 Primers used for PCR of endogenous virus-like regions of some animal genomes.

Target species	Primer name	Primer sequence	Genomic location	product size (bp)
Canis lupus familiaris (dog)	Dog_chr5F	5'-TTTGATGTGCCAGTATACAATTTGTGGG-3'	NC_006587.2 chr5: 33114170-33114197	951
	Dog_chr5R	5'-GGAAGCTGGTCTGGACTGTACACCATA-3'	NC_006587.2 chr5: 33115094-33115120	
	Dog_chr15F	5'-TTCAGCTCAGGTCATCATCTCAGGGTC-3'	NC_006597.2 chr15: 58042540-58042566	959
	Dog_chr15R	5'-GCAGAGGAAACAAGTGCAAAGGGTCT-3'	NC_006597.2 chr15: 58043473-58043498	
	Dog_chr17F	5'-GCCAACAAATGGAGACTGGAGGAACA-3'	NC_006599.2 chr17: 37420265-37420290	767
	Dog_chr17R	5'-GCGCGACTAGGCAGGAATAGTAGTAAGA-3'	NC_006599.2 chr17: 37421004-37421031	
	Dog_chr22F	5'-CTCCGTTTGCTCGGACATTTCCATAA-3'	NC_006604.2 chr22: 64349628-64349653	990
	Dog_chr22R	5'-AAAGGGCCTCCTACCCCAGACTTCAA-3'	NC_006604.2 chr22: 64350592-64350617	
Felis catus (domestic cat)	Cat_071F	5'-GACAATGGCTGAGAATGAACAAAGTGAA-3'	ACBE01020071.1: 530-557	1003
	Cat_071R	5'-GCTTAAGCTAAGAATCACTGGAGGACACT-3'	ACBE01020071.1: 1504-1532	
	Cat_274F	5'-CCAGCAATTAGAACAAAGTCACACAAACCA-3'	ACBE01129274.1: 1-30	629
	Cat_274R	5'-TTCCTTGAAAGACTGTGAACCTTATCCTGT-3'	ACBE01129274.1: 600-629	
	Cat_791F	5'-GCAGGACTGACTAGGACAATAAAAGGAA-3'	ACBE01511791.1: 563-590	975
	Cat_791R	5'-GACTATGGAACTCACTACAGGTAGCAAAAG-3'	ACBE01511791.1: 1508-1537	

Chromosomal locations and matching PCR primers are provide
